# Multicentric Validation of Proteomic Biomarkers in Urine Specific for Diabetic Nephropathy

**DOI:** 10.1371/journal.pone.0013421

**Published:** 2010-10-20

**Authors:** Alaa Alkhalaf, Petra Zürbig, Stephan J. L. Bakker, Henk J. G. Bilo, Marie Cerna, Christine Fischer, Sebastian Fuchs, Bart Janssen, Karel Medek, Harald Mischak, Johannes M. Roob, Kasper Rossing, Peter Rossing, Ivan Rychlík, Harald Sourij, Beate Tiran, Brigitte M. Winklhofer-Roob, Gerjan J. Navis, for the PREDICTIONS Group

**Affiliations:** 1 Department of Internal Medicine, University Medical Center Groningen, University of Groningen, Groningen, The Netherlands; 2 Diabetes Centre, Isala Clinics, Zwolle, The Netherlands; 3 Mosaiques diagnostics GmbH, Hannover, Germany; 4 Department of General Biology and Genetics, Third Faculty of Medicine, Charles University, Prague, Czech Republic; 5 Institute of Human Genetics, Medical Faculty Heidelberg, University of Heidelberg, Heidelberg, Germany; 6 Human Nutrition & Metabolism Research and Training Center, Institute of Molecular Biosciences, Karl Franzens University of Graz, Graz, Austria; 7 Faculty of Medicine, BHF Glasgow Cardiovascular Research Centre, University of Glasgow, Glasgow, United Kingdom; 8 Clinical Division of Nephrology and Hemodialysis, Department of Internal Medicine, Medical University of Graz, Graz, Austria; 9 Steno Diabetes Center, Gentofte, Denmark; 10 Second Department of Internal Medicine, Third Faculty of Medicine, Charles University, Prague, Czech Republic; 11 Division of Endocrinology and Nuclear Medicine, Department of Internal Medicine, Medical University of Graz, Graz, Austria; 12 Clinical Institute of Medical and Chemical Laboratory Diagnostics, Medical University of Graz, Graz, Austria; University of Tor Vergata, Italy

## Abstract

**Background:**

Urine proteome analysis is rapidly emerging as a tool for diagnosis and prognosis in disease states. For diagnosis of diabetic nephropathy (DN), urinary proteome analysis was successfully applied in a pilot study. The validity of the previously established proteomic biomarkers with respect to the diagnostic and prognostic potential was assessed on a separate set of patients recruited at three different European centers. In this case-control study of 148 Caucasian patients with diabetes mellitus type 2 and duration ≥5 years, cases of DN were defined as albuminuria >300 mg/d and diabetic retinopathy (n = 66). Controls were matched for gender and diabetes duration (n = 82).

**Methodology/Principal Findings:**

Proteome analysis was performed blinded using high-resolution capillary electrophoresis coupled with mass spectrometry (CE-MS). Data were evaluated employing the previously developed model for DN. Upon unblinding, the model for DN showed 93.8% sensitivity and 91.4% specificity, with an AUC of 0.948 (95% CI 0.898-0.978). Of 65 previously identified peptides, 60 were significantly different between cases and controls of this study. In <10% of cases and controls classification by proteome analysis not entirely resulted in the expected clinical outcome. Analysis of patient's subsequent clinical course revealed later progression to DN in some of the false positive classified DN control patients.

**Conclusions:**

These data provide the first independent confirmation that profiling of the urinary proteome by CE-MS can adequately identify subjects with DN, supporting the generalizability of this approach. The data further establish urinary collagen fragments as biomarkers for diabetes-induced renal damage that may serve as earlier and more specific biomarkers than the currently used urinary albumin.

## Introduction

Diabetic nephropathy (DN) is a leading cause of morbidity and mortality in patients with diabetes mellitus [1]. Accurate diagnostic tools are important, not only for the allocation of preventive measures but also to better unravel the complex pathogenesis of DN. Current clinical biomarkers used to diagnose diabetic kidney disease, urinary albumin excretion and glomerular filtration rate, are subject to considerable measurement variability [2], and are heterogeneous as to prognostic impact [3]. Whereas albuminuria is broadly used as a renal biomarker, its specificity is still subject of debate [4]. Moreover, urinary albumin excretion and glomerular filtration rate (GFR) are also affected in non-diabetic renal disease, and accordingly not specific for diabetic nephropathy [5]. As such their potential to detect and monitor the specific pathogenetic processes involved in diabetic nephropathy is limited. Furthermore, especially GFR, but also albuminuria are late stage biomarkers, only indicative after substantial organ damage [6]. Alternative non-invasive diagnostic methods, that may enable detection of DN at an earlier stage, and/or with higher accuracy, would be beneficial for clinical management of diabetic patients, as well as for pathogenetic studies aimed at further deciphering pathophysiology, and identifying targets for intervention. Potential sources for such biomarkers may be urinary proteins and/or peptides, as these should display significant changes at an early state of disease, displaying initial pathophysiological changes in the kidney [7].

Proteome analysis using capillary electrophoresis coupled mass spectrometry (CE-MS) has recently emerged as a powerful tool to define biomarkers that enable diagnosis [8], [9], prognosis [10], assessment of therapeutic intervention [11], and monitoring of specific pathogenetic pathways. The different technological considerations, both with respect to samples and technological platform have recently been discussed and reviewed [12]–[15]. We have focused on urinary proteome analysis as the urinary proteome has been found to be quite stable [16], [17] and contains an array of low molecular weight proteins and peptides that can be analyzed without the need for additional manipulation such as proteolytic digests [18].

Recent studies demonstrated that urinary proteome analysis enables the definition of biomarkers specific for chronic kidney disease (CKD) [12], [19] and for DN [1], [20]. These might prove valuable in clinical practice. As a first step, however, confirmation of the diagnostic value of these markers in a controlled study in independent clinical centers, different from the ones that were involved in the identification of the biomarkers, has to be obtained. Such rigid independent confirmation is required prior to any further development, investigating e.g. prognostic value, to clearly support the validity and reliability of the biomarkers and biomarker-based models [21]. In the past, confirmation of potential disease-associated biomarkers has often failed (e.g. [22], [23]), hence this step is of the outmost importance. Therefore, we aimed to validate identified biomarkers and a biomarker-based model for DN that was described previously in an independent blinded set of samples [1], collected prospectively in multiple centers not involved in the original identification of biomarkers to rule out any center-based bias.

To ease data interpretation, a case-control set-up was chosen. The low molecular proteome of diabetes mellitus type 2 patients with normoalbuminuria (controls) and matched diabetes patients with diabetic nephropathy (cases) was analyzed in a blinded study (PREDICTIONS study) by capillary electrophoresis-mass spectrometry (CE-MS), and samples were successful classified using the previously defined biomarker model.

## Methods

### Ethics Statement

The study was conducted according to the requirements of the Declaration of Helsinki, the protocol was approved by the respective Ethical review boards of the participating centers (Medical Ethics Committees of the Isala Clinics in Zwolle and of the University Medical Center in Groningen, the Ethics Committee of Third Faculty of Medicine, Charles University at Prague, and the Ethics Committee of the Medical University of Graz), and written informed consent was obtained from all patients.

### Settings and participants

The study was set up as a cross-sectional case-control study, cases being type 2 diabetes patients with nephropathy, controls being type 2 diabetes patients without nephropathy. Patients aged 35–75 with type 2 diabetes with a documented duration of diabetes of ≥5 years were eligible. Diagnosis of diabetes was established in accordance with the WHO criteria, by the following: fasting plasma glucose ≥7.0 mmol/l, a two-hour value in an oral glucose tolerance test ≥11.1 mmol/l, or random plasma glucose ≥11.1 mmol/l in the presence of symptoms. Type 2 diabetes was diagnosed by lack of criteria for type 1 diabetes. Inclusion criteria for cases were: albuminuria >300 mg/d and known overt diabetic retinopathy. Retinopathy is requested to be present is to ensure that albuminuria is the consequence of diabetic nephropathy rather than a non-diabetic glomerulopathy. A renal biopsy would be the gold standard to discriminate between diabetic nephropathy and a non-diabetic glomerulopathy, but a renal biopsy is nearly never taken in diabetic patients and several studies have indicated that the request for retinopathy being present is a good alternative for discrimination between diabetic nephropathy and non-diabetic glomerulopathy in type 2 diabetic patients with albuminuria [24]–[26]. Exclusion criteria were end stage renal failure, known causes of renal failure other than diabetes and non-Caucasian ethnic origin. Controls were matched within center for gender and diabetes duration. Exclusion criteria for controls were micro-albuminuria, non-Caucausian ethnic origin, and in case of use of RAAS-blocking medication, unknown albuminuria status prior to start of treatment. Patients were prospectively recruited from the outpatient clinics for diabetes and nephrology in three participating centers, located in Zwolle (The Netherlands), Graz (Austria), and Prague (Czech Republic), respectively.

### Sample collection and preparation

The second urine of the morning was collected as described [27] and stored frozen below −20°C. A 0.7 mL aliquot was thawed immediately before use and diluted with 0.7 mL 2 M urea, 10 mM NH_4_OH containing 0.02% SDS. In order to remove high molecular weight polypeptides, samples were filtered using Centrisart ultracentrifugation filter devices (20 kDa molecular weight cut-off; Sartorius, Goettingen, Germany) at 3,000 g until 1.1 mL of filtrate was obtained. Subsequently, filtrate was desalted using PD-10 column (GE Healthcare, Sweden) equilibrated in 0.01% NH_4_OH in HPLC-grade water. Finally, samples were lyophilized and stored at 4°C. Shortly before CE-MS analysis, lyophilisates were resuspended in HPLC-grade water to a final protein concentration of 0.8 µg/µL checked by BCA assay (Interchim, Montlucon, France).

### CE-MS analysis

CE-MS analysis was performed as described [27], using a P/ACE MDQ capillary electrophoresis system (Beckman Coulter, Fullerton, USA) on-line coupled to a Micro-TOF MS (Bruker Daltonic, Bremen, Germany). Data acquisition and MS acquisition methods were automatically controlled by the CE *via* contact-close-relays. Spectra were accumulated every 3 s, over a range of *m/z* 350 to 3000 Th. Accuracy, precision, selectivity, sensitivity, reproducibility, and stability are described in detail elsewhere [27], [28]. The average recovery of sample in the preparation procedure was ∼85% and the limit of detection was ∼1 fmol. Mass resolution was above 8,000 enabling resolution of monoisotopic mass signals for z≤6. After charge deconvolution, mass accuracy was <25 ppm for monoisotopic resolution and <100 ppm for unresolved peaks (z>6). The analytical precision of the set-up was assessed by (a) reproducibility achieved for repeated measurement of the same replicate and (b) by the reproducibility achieved for repeated preparation and measurement of the same urine sample. To ensure high data consistency, a minimum of 950 peptides/proteins had to be detected with a minimal MS resolution of 8,000 in a minimal migration time interval of 10 minutes.

### Data processing

Mass spectral ion peaks representing identical molecules at different charge states were deconvoluted into single masses using MosaiquesVisu software [29]. Both CE-migration time and ion signal intensity (amplitude) show variability, mostly due to different amounts of salt and peptides in the sample and are consequently normalized. Reference signals of 1770 urinary polypeptides are used for CE-time calibration by local regression. For normalization of analytical and urine dilution variances, MS signal intensities are normalized relative to 29 “housekeeping” peptides generally present in at least 90% of all urine samples with small relative standard deviation, as described in detail recently [28]. For calibration, local regression is performed. The obtained peak lists characterize each polypeptide by its molecular mass [Da], normalized CE migration time [min] and normalized signal intensity. All detected peptides were deposited, matched, and annotated in a Microsoft SQL database allowing further statistical analysis.

### Classification model of DN

Data of the current samples were tested against the previously developed biomarker model for DN [1]. Rossing et al. defined and validated models for the differentiation of diabetic patients type 1 with macroalbuminuria and normoalbuminuria after CE-MS analysis. Among these diabetes patients, 102 urinary biomarkers differed significantly between patients with normoalbuminuria and DN. For reduction of the number of variables, a “take-one-out” procedure was used, decreasing the number of biomarkers to 65 without losing performance in the classification. A support vector machine (SVM) biomarker model with these 65 polypeptides identified patients with DN in blinded data set of 70 individuals (35 cases and 35 controls) with 100% sensitivity and 97% specificity (AUC = 0.994).

SVM-based classification on the urinary peptidome was performed using MosaCluster software (version 1.7.0) [30]. This software tool allows the classification of samples in the high-dimensional parameter space by using support vector machine (SVM) learning. For this purpose, MosaCluster generates polypeptide models, which rely on polypeptides displaying statistically significant differences when comparing data from patients with a specific disease to controls or other diseases, respectively. Each of these polypeptides represents one dimension in the n-dimensional parameter space [9], [31]–[33]. SVM views a data point (probands plasma sample) as a p-dimensional vector (p numbers of protein used), and it attempts to separate them with a (p-1) dimensional hyperplane. There are many hyperplanes that might classify the data. However, maximum separation (margin) between the two classes is of additional interest, and therefore, the hyperplane with the maximal distance from the hyperplane to the nearest data point is selected. All marker proteins are used without any weighting to build up the n-dimensional classification space and to display the data set in the classification space. Classification is performed by determining the Euclidian distance of the data set to the n-1 dimensional maximal margin hyperplane (absolute value of the normal vector) and the direction of the vector (class 1 or class 2).

### Statistical analysis

Sensitivity and specificity of the previously defined biomarker models, and 95% confidence intervals (95% CI) were calculated using receiver operating characteristic (ROC) plots (MedCalc version 8.1.1.0, MedCalc Software, Belgium, www.medcalc.be) [34]. Furthermore, Mann-Whitney test (for independent samples) was performed to receive Box-Whisker-Plots with this software. Statistical significance was assumed at p<0.05. For analysis of differences of individual peaks between cases and controls, statistical significance was assumed at p<0.001 to account for multiple testing. For the correlation analysis of each peptide biomarker, Rank correlation was used with Spearman's rank correlation coefficient (Spearman's ρ) (MedCalc version 8.1.1.0, MedCalc Software, Belgium, www.medcalc.be). For biomarker definition, polypeptides that were found in more than 70% of the samples in at least one of the two groups (DN or non-DN) were considered. This pre-defined set of polypeptides was further validated by randomly excluding 30% of available samples. This bootstrapping procedure was repeated up to 10 times. Further on, mutilvariate statistic methods (e.g., Benjamini-Hochberg) were applied for selection refinement.

### Sequencing of peptides

Candidate biomarkers from urine were sequenced using CE-MS/MS or LC-MS/MS as recently described [35].

**Figure 1:**
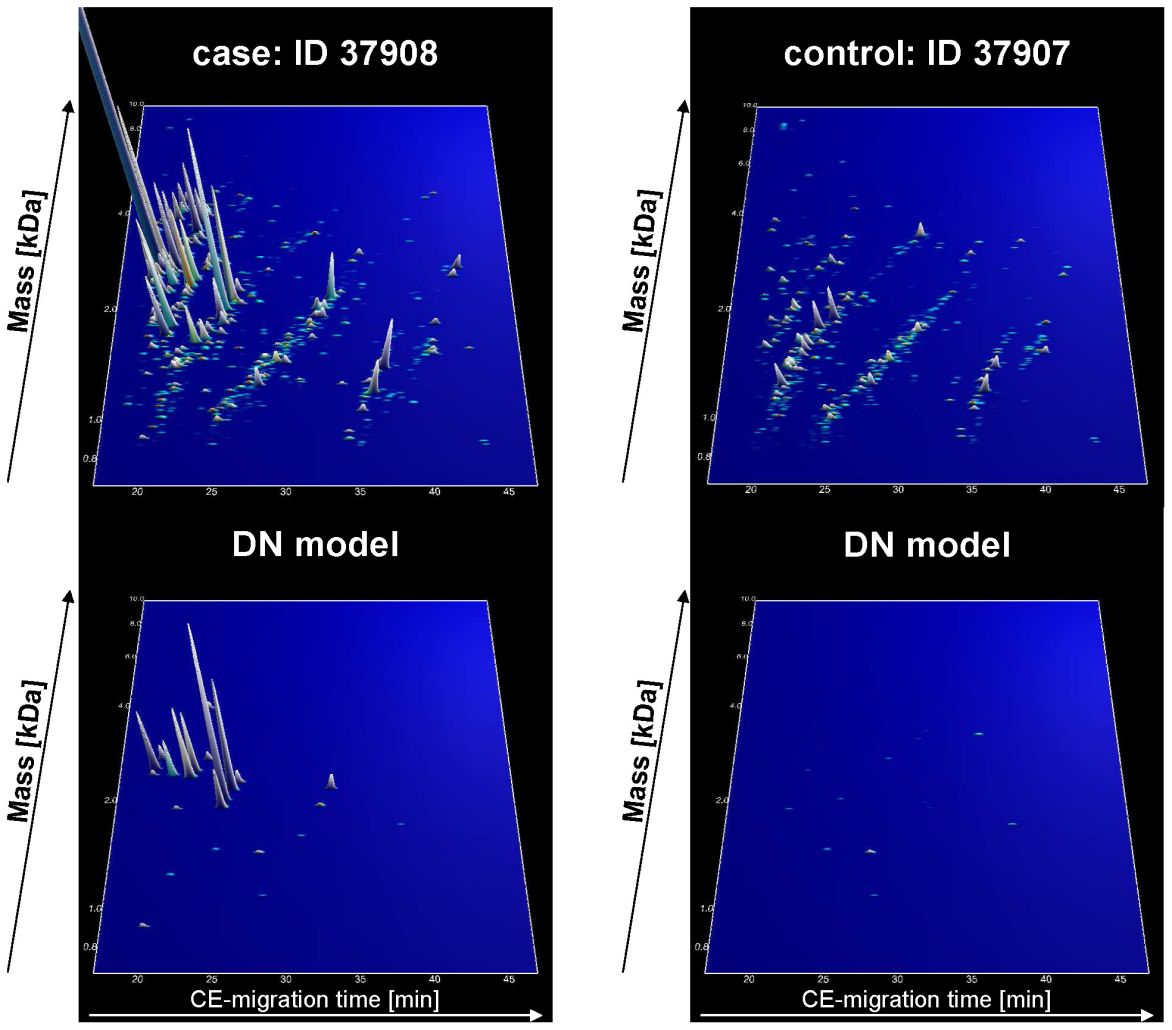
Polypeptide patterns of exemplarily urine samples. The upper panel shows polypeptide patterns of all peptides, which are in the urinary proteome from one patient with (ID: 37908) and one patient without DN (ID: 37907). The lower panel shows distinct peptides of the DN model of these patients urine sample. Each polypeptide is defined by its CE-migration time (x-axis, minutes), mass (y-axis, kDa), and signal intensity (z-axis). The molecular mass is indicated on the left, the normalized migration time is indicated on the bottom.

Raw data files were either converted into dta-files (RAW files generated by ion traps from Thermo Fisher Scientific) with the use of DTA Generator [36], [37] or into mgf-files (data derived from MALDI-TOF and Q-TOF analyses) with the use of DataAnalysis (version 4.0; Bruker Daltonik). All resultant MS/MS data were submitted to MASCOT (www.matrixscience.com; release number: 2.3.01) for a search against human entries (20,295 sequences) in the Swiss-Prot database (Swiss-Prot number 2010.06) without any enzyme specificity and with up to one missed cleavage. No fixed modification was selected, and oxidation products of methionine, proline, and lysine residues were set as variable modifications. Accepted parent ion mass deviation was 0.5 Da (20 ppm for all Orbitrap spectra); accepted fragment ion mass deviation was 0.7 Da. Only search results with a MASCOT peptide score equally or higher as the MASCOT score threshold were included (see **table S3a**). Additionally, ion coverage was controlled to be related to main spectral fragment features (b/y or c/z ion series) (see also **figure S1**). For further validation of obtained peptide identifications, the strict correlation between peptide charge at pH 2 and CE-migration time was utilized to minimize false-positive identification rates [38],[39]. As depicted in **figure 1**, the polypeptides are arranged in four to five lines. The members of each line are characterized by the numbers of basic amino acids (arginine; histidine; lysine) included in the peptide sequence. Specifically, the peptides in the right line contain no basic amino acids, only the N-terminus of the peptide is positively charged at pH 2. In contrast, peptides in the other lines (from right to left) show increasing number of basic amino acids in addition to their N-terminal ammonium group [39]. Calculated CE-migration time of the sequence candidate based on its peptide sequence (number of basic amino acids) was compared to the experimental migration time. A peptide was accepted only if it had a mass deviation below ±50 ppm and a CE-migration time deviations less than ±2 min.

## Results

The case/control study was composed of 148 diabetes mellitus type 2 patients, including 65 cases and 83 controls. The patients were well-matched for age, gender and diabetes duration. Blood pressure was significantly higher, and creatinine clearance significantly lower in cases (all p<0.05). Albuminuria was by definition present in cases, and absent in control. All samples were analyzed with CE-MS. For 145 urine samples (64 cases and 81 controls) of this study population high quality CE-MS data sets were obtained. The data obtained from 3 samples did not pass quality control and were excluded from the subsequent analysis. Patient characteristics and classification scores of the proteome analysis are presented in **table 1**. The data on all relevant peptides detected in the 145 samples are listed in **table S1**.

**Table 1:** Patient characteristics (means ± SD) of the PREDICTIONS cohort.

	n	M/F	Age [year]	Duration DM [yr]	SBP [mmHg]	DBP [mmHg]	UAE [mg/L]	CrCl [ml/min/1.73 m^2^]
*cases*	64	44/20	64±10	17±8	143±21	78±12	953±931	72±40
*controls*	81	47/34	62±11	16±6	133±15	74±11	6±4	94±32

M/F: male/female ratio; SBP: systolic blood pressure; DBP: diastolic blood pressure; UAE: urinary albumin excretion; CrCl: creatinine clearance estimated by Cockroft-Gault equation.

Rossing et al. defined and validated models for the differentiation of diabetic patients type 1 with macroalbuminuria and normoalbuminuria after CE-MS analysis [1]. A support vector machine biomarker model (SVM-BM) composed of 65 of these biomarkers identified DN in blinded data set with 100% sensitivity and 97% specificity. This ‘DN model’, was applied to the collected ‘case and control’ samples of the PREDICTIONS study cohort. The scoring of all individual samples using the biomarker model is given in **table S2**. After evaluation of the blinded samples, all data were reported to the central study database for further evaluation and subsequent unblinding. After unblinding, accuracy of prediction was assessed. The complete polypeptide profiles and the DN-specific panels are depicted in **figure 1** exemplarily for one patient with and one without DN.

**Figure 2:**
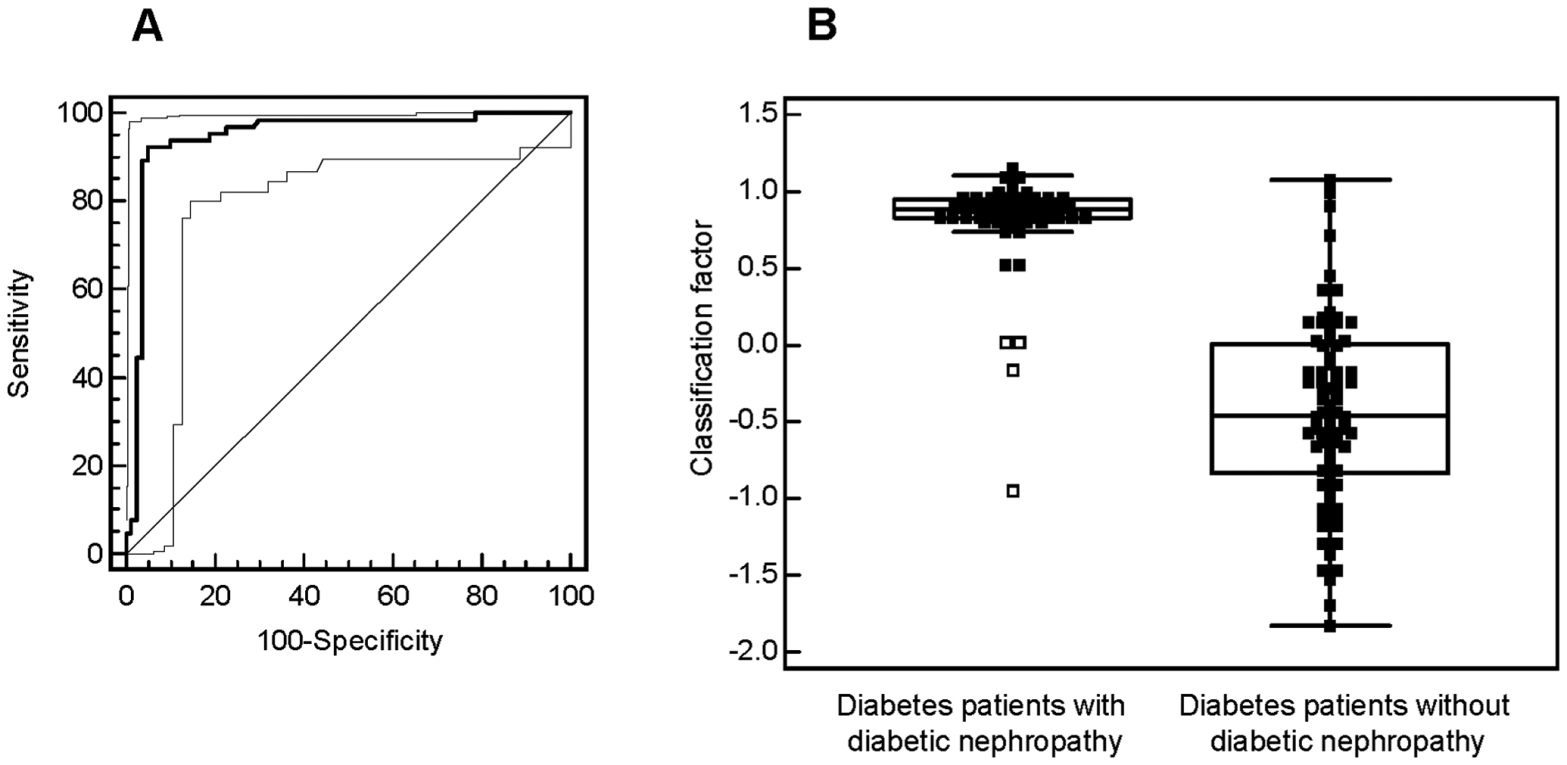
Statistical analysis of the classification results. **A**) ROC curve and **B**) Box-Whisker-plot for classification of the ‘case and control’ patient collective with the ‘DN’ pattern are shown.

Classification of the ‘case/control’ urine samples with this ‘DN biomarker model’ was accomplished with sensitivity of 93.8% and specificity of 91.4%. The AUC value in the ROC-analysis was 0.948 [95% CI: 0.898 to 0.978] (see **figure 2A**). As depicted in the Box-and-Whisker plot in **figure 2B** **,** this classification resulted in a significant (P<0.0001) difference of the median classification factor between patients with DN (0.889 [95% CI: 0.843 to 0.924]) and patients without DN (−0.461 [95%°CI: −0.592 to −0.255]). The classification results are shown in **table S2** (‘DN model’).

Of the 65 previously defined differentially expressed peptides [1], in the current study 92.3% (60 markers) could be confirmed as being significantly different in this PREDICTIONS cohort between diabetic patients with DN and diabetic controls at p<0.05 and 50/60 were significant at p<0.0001. The results of the statistical analysis are listed in **table S3a**.

**Figure 3:**
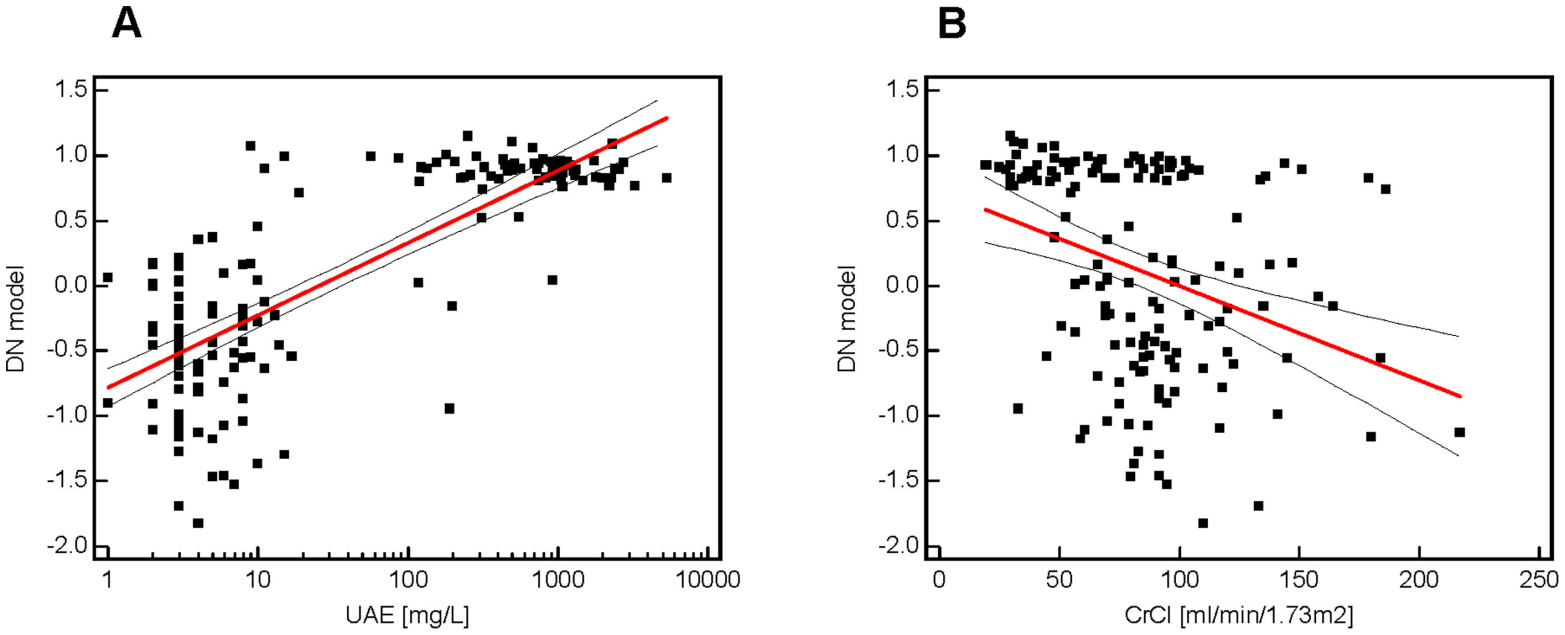
Correlation analysis. Scatter diagrams of correlation from proteomic biomarker pattern with urinary albumin excretion (UAE) (**A**) and creatinine clearance (**B**). The red line shows the regression line with 95% confidence interval (dashed line).

A correlation analysis of these classification factors and the clinical parameters was performed (see **figure 3**). In **figure 3A** the correlation with log urinary albumin excretion (UAE) is demonstrated with a positive correlation coefficient r = 0.701 [95% CI: 0.607 to 0.775] and a significance level of P<0.0001. Here, most of the urine samples from patients without DN (<20 mg/L) have classification factors below 0.3 and from patients with DN (<200 mg/L) have classification factors above 0.5. The correlation analysis of the proteomic results with the creatinine clearance (CrCl) (see **figure 3B**) resulted in a negative correlation (r = −0.368 [95% CI: −0.501 to −0.218] (P<0.0001)), which is lower than the correlation with urinary albumin excretion. Many patients with a classification factor above 0.5 also show a CrCl <90 ml/min/1.73 m^2^. Otherwise, patients with classification factors lower than 0.5 differ in their CrCl between 50–200 ml/min/1.73 m^2^.

**Figure 4:**
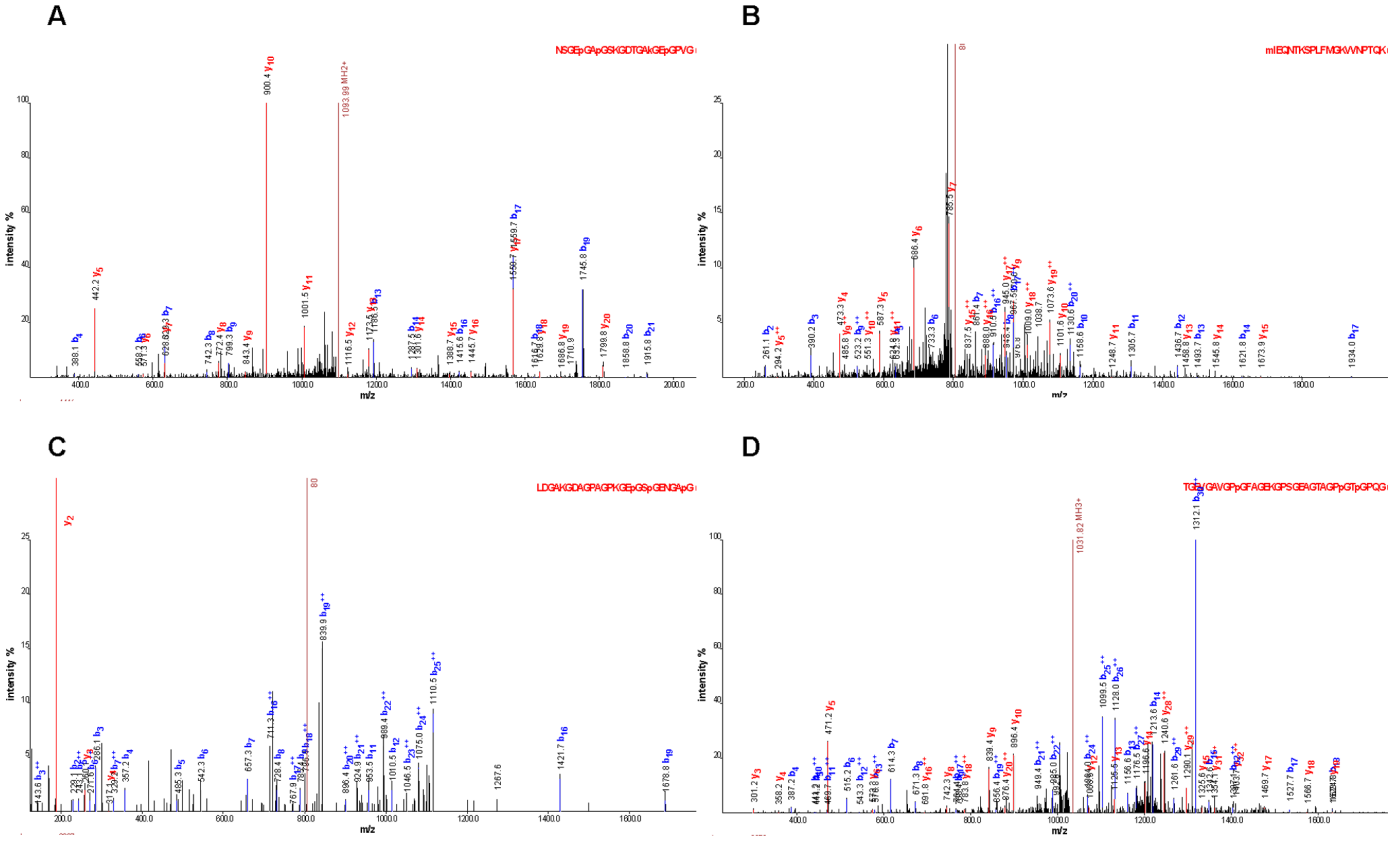
Annotated fragment spectra of new identified peptides. Four MS/MS spectra are shown exemplarily of the new identified DN biomarkers, like collagen alpha-1 (I) fragments (**A, C**), alpha-1-antitrypsin fragment (**B**), and a collagen alpha-2 (I) fragment (**D**). The corresponding sequence is displayed above each spectrum (p = hydroxylation of P; k = hydroxylation of K; m = oxidation of M). Identified b-ions are marked in blue and y-ions in red color.

**Figure 5:**
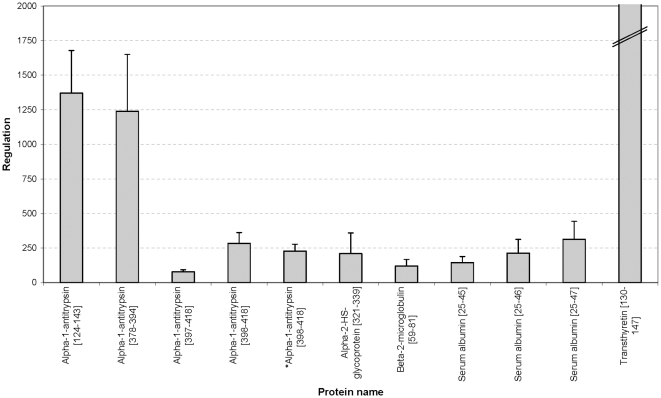
Up-regulation of blood derived protein fragments in urine samples of the PREDICTIONS cohort. Displayed is the regulation of alpha-1-antitrypsin fragments, an alpha-2-HS glycoprotein fragment, a beta-2-microglobulin fragment, serum albumin fragments, and a transthyretin fragment. The asterisk (*) indicate same peptide with one more modification (oxidation).

**Figure 6:**
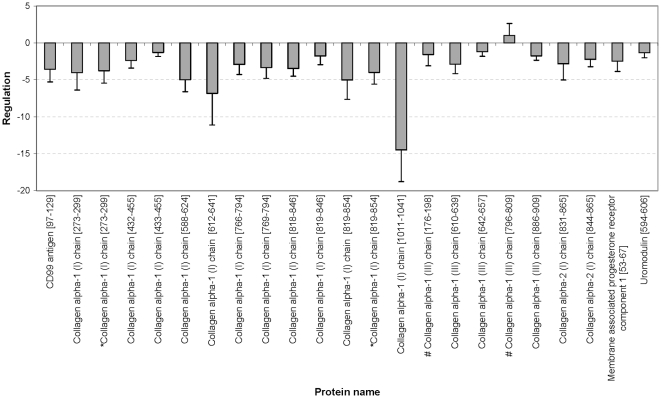
(Down-) Regulation of further peptide markers in urine samples of the PREDICTIONS cohort. Displayed is the regulation of a CD99 antigen fragment, different collagen fragments, membrane associated progesterone receptor component 1 fragment, and uromodulin fragment. The hash (#) depicts peptide fragments, which are not significant in this cohort. The asterisk (*) indicate same peptide with one more modification (hydroxylation).

As depicted in **table S3a**, 34 of the 65 biomarkers could be sequenced until today. We have identified 8 more peptides in comparison to in the previous study [1], where the used biomarker pattern was generated. The annotated fragment spectra of these new peptides are depicted in **figure 4** (a summary of all annotated fragment spectra are shown in **figure S1**). For the sequenced peptides the direction of regulation is illustrated in **figure 5** **and** **6**. Up-regulated markers in urine samples of patients with DN (see **figure 5** **)** are fragments of blood components, like alpha-1-antitrypsin, albumin, transthyretin, alpha-2-HS-glycoprotein, and beta-2-microglobulin. In **figure 6** the regulation of CD99 antigen fragment, collagen fragments, membrane associated progesterone receptor component 1 fragment, and uromodulin fragment is shown. Only one collagen fragment is up-regulated in urine samples of DN patients. This peptide belongs to the five biomarkers, which are not significant different between cases and controls (see above and **table S3a**).

In addition, a Rank correlation of each of the 65 biomarker was performed. The results (p-values and correlation coefficient) are shown in **table S4**. All biomarkers, which presented a significant correlation, have reciprocal correlation coefficients for UAE and CrCl. As expected the correlation between albumin excretion and known albumin and other blood protein fragments is positive, in contrast to the correlation of albumin excretion with the collagen fragments, CD99 antigen, membrane-associated progesterone receptor component 1, and uromodulin fragments. Furthermore, the correlation of UAE with blood protein fragments is stronger than the correlation with collagen fragments. The five biomarkers, which are not significant in the U-test (see **table S3a**), also show no significant correlation to urinary albumin excretion. The Rank correlation of creatinine clearance with collagen fragments and blood protein fragments is not very high in both cases.

Investigation of the false positive classified patients indicated that in several cases the classification “diabetic renal damage” may in fact be correct, but albuminuria may be under the respective criteria (see method section: ‘Methods’). Seven ‘control’ patients were classified as cases. All of them show GFR values below 90 (stage 2), three of them even have a GFR<60 (stage 3), and two of them have an increasing urinary albumin excretion at a later visit (approximately 1 year later). These data may indicate the utility of the biomarkers not only for detection of overt nephropathy, but also prediction of its development in patients with diabetes and normoalbuminuria.

**Figure 7:**
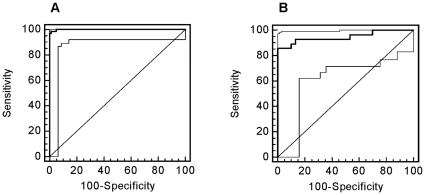
ROC curves for classification of the patient collectives with the ‘DN type 2’ pattern. ROC analysis for CKD diagnosis of the training set (**A**) and the test set (**B**).

For the generation of a new model for DN in diabetic type 2 patients (using the PREDICTIONS cohort), urinary polypeptides of the control group were compared with those of patients with diabetic nephropathy. This analysis identified 103 peptides of statistical significance using multivariate statistic analysis like Benjamini-Hochberg [40] (p* = *0.05; see **table S3b**). A support vector machine-based model with these biomarkers discriminated controls from cases with 98% sensitivity and 99% specificity. The distribution of the polypeptides in the two groups is shown in **figure 7A**. The validity of the ‘DN type 2’ biomarkers was further evaluated in a diabetes type 1 test-set cohort (trainingset of Rossing et al. [1]) and resulted in 86% sensitivity and 100% specificity with an AUC value of 0.948 (see **figure 7B**). Of the 103 defined differentially expressed peptides, 65% (67 markers) could be confirmed as also being significantly different in the ‘Rossing’ cohort between diabetic patients with DN and diabetes controls. The results of the statistical analyses are listed in **table S3b**. Of note, most biomarkers with high significance were found to be significantly different in both cohorts.

## Discussion

This study provides independent confirmation of the performance of a previously developed biomarker model for diabetic nephropathy using proteomic analysis with high-resolution CE-MS of the urinary proteome [1], [41]. The model for DN has high specificity and sensitivity, notwithstanding the fact that the current study was done in subjects with type 2 diabetes, whereas the population used for development of the model for diagnosis of diabetic nephropathy had diabetic mellitus type 1. These findings clearly indicate that the applied peptide pattern allowed diabetes-type independent classification of diabetic nephropathy.

For most of the peptides in the ‘DN model’, the difference between the cases and controls reached a high level of statistical significance, with p-values <0.0001, demonstrating the high selectivity of the urinary proteome analysis. The fact that 92% of the markers included in the ‘DN model’ were also significant in the PREDICTIONS cohort supports a valid strategy of marker selection in the Rossing study [1].

When distinguishing patients with DN from normoalbuminuric diabetic patients, the distribution of the classification factors in the control group (patients without DN) was broader than in the case group (see **figures 2B** **and** **3A**). This may be explained by the existence of early stages of DN, in the absence of any clinical symptoms yet. The arrangement of the case and control group was performed based on classical urine analyses (urinary albumin excretion rate).

Identification of the specific peptides in the biomarker model may allow better insights in patho-physiological pathways involved in renal damage in general, and specific pathways for renal damage in diabetes. The regulation of the sequenced biomarkers in the ‘DN model’, as reported here, shows a consistent pattern that is apparently specific for DN. The up-regulation of the serum protein fragments and the down-regulation of the collagen fragments in the urine is a consistent feature of DN, as also discussed in detail in the literature (see [6]). Furthermore, the correlation analysis confirms these findings. As expected, the correlation of the biomarkers with UAE and with CrCl resulted in reciprocal values.

Thousand-fold up-regulation of blood-derived protein fragments in urine (see **figure 3B**) is expected in the light of substantial glomerular damage that results in albuminuria. Hence, this likely does not reflect better or earlier markers. The presence of high amounts of these proteins likely indicates an insufficiency of readsorption or altered glomerular permeability of the kidney, implying an existing damage. In contrast, changes in the collagen metabolism may be closely linked to early renal damage in patients with diabetes and may help to provide information for the prognosis and monitoring of DN [1]. Type I and III collagens alpha-1 are main components of renal interstitial fibrosis [42]. It is tempting to speculate that the decrease in urinary collagen fragments reflects decreased collagen breakdown, and hence a propensity to progressive fibrotic lesions. However, the origin of the urinary collagen fragments cannot be determined from the current data and must be investigated in further studies. The differential excretion of uromodulin fragments gains additional interest from the recently reported association between genetic variation in the coding for uromodulin with susceptibility to CKD [43]. Finally, altered collagen metabolism appears to be involved in non-diabetic CKD as well, albeit with a differential excretory pattern. The patho-physiological impact of this finding deserves further exploration.

Investigation of the few cases where the classification factors of the DN model did not coincide with the clinical diagnosis suggests that several of these may in fact not be incorrectly classified by the DN model, but the clinical assessment of the patient was not correct (see also **figure 3A**: classification factor >0.3 and UAE <10 mg/L). Of the 7 controls that were classified as cases, all patients had at least GFR below 90, indicating the presence or the possible onset of renal disease. It is tempting to speculate that these patients may well have developed a diabetic renal disease that does not exactly resemble “classical DN”, hence is undetected by assessing albuminuria only.

The generation of a new model for DN with type 2 diabetic patients (PREDICTINS cohort) and the validation of this model with type 1 diabetic patients (Rossing cohort), resulted in the same AUC value as the validation of the previously defined ‘DN type 1’ model with the PREDICTIONS cohort. The differences in the biomarker selection/identification in type 1 and 2 diabetic patient urine samples may be caused by different patho-physiology of the DN, but also by the differences in both cohorts in general. The groups differ in age (∼10 years), diabetes duration (∼20 years), and medication (e.g. insulin). Therefore, this study is not suited for the analysis of differences of DN derived from type 1 or 2 diabetes. Also, the investigation of such potential differences was not a focus of this study. This will require greater population sizes and external validation of training profiles and components of profiles in independent populations of subjects with type 1 and type 2 diabetes and should be the focus of a further study.

In conclusion, urinary profiling using CE-MS was successfully applied to urine samples of an independent population of diabetic patients with or without existing DN. A biomarker model for the identification of patients with DN was validated with this multicenter blinded test set and allowed diagnosis of DN with high accuracy. These results provide clear independent confirmation for the accuracy of urinary proteome analysis for detection of DN. As these biomarkers have now been validated in independent clinical centers, we will in the next step investigate their prognostic value. Albumin excretion does reflect late pathological changes. However, we are tempted by the data presented to speculate that the assessment of urinary collagen fragments may result in a substantial improvement, enabling detection of diabetic nephropathy at earlier stages. This is also indicated by preliminary data on small populations ([1] and unpublished). As a next step, these promising data have to be verified in longitudinal studies of sufficient statistical power to prove, or disprove, the prognostic value of the urinary collagen fragments.

## Supporting Information

Figure S1 The mass spectra annotated with fragment assignments of all identified peptides (see Protein ID) from the Mascot searches are shown.
(0.28 MB PPT)

Table S1 Raw data and additional information. Table consists of 3 different spreadsheets called polypeptides, patients ID, and patient's raw data. Polypeptides. Table listing all 5,616 different peptides/proteins (Protein ID) detected, their calibrated molecular mass [Da], and normalized CE migration time [min]. Patient ID. Table includes all 145 patients. Patients ID correlated to their sampling center and original ID. Patient's raw data. Tables in pivot format show the CE-MS data of the 145 samples in the database. The protein IDs of all peptides are given in the first column named “Protein ID”; the unique patients IDs constitute the first row. The MS data from each sample are reflected in one column. The number in each cell represents the calibrated amplitude of the mass spectrometric signal of each peptide/protein detected in the sample.
(6.60 MB XLS)

Table S2 Patient characteristics. This table lists information of each patient's sample, including clinical center, original ID, gender, age, GFR, and albuminuria. Furthermore, the classification factors of the DN model and the diagnoses are listed.
(0.04 MB XLS)

Table S3 (a) The 65 previously defined urinary DN biomarkers that were used in the DN type 1 biomarker model [1] and their distribution in the “PREDICTIONS” study. Shown are the protein identification number in the data set (ID), mass (in Dalton), and normalized CE-migration time (in minutes), the p-values (U-test; significant biomarkers are marked in bold letters), and the mean amplitudes with standard deviations (SD) in the two groups, diabetic patients with and without diabetic nephropathy. In addition, sequences (modified amino acids: p = hydroxyproline; k = hydroxylysine; m = oxidized methionine), protein names, Swiss-Prot entries, calculated masses [Da], and the difference between CE-MS measured and calculated masses [ppm] are shown for the identified markers. In the next five columns information on MS/MS database search are given (best Mascot score; observed m/z mass; charge of the precursor ion; differences between measured LC-MS mass and calculated mass; used device and dissociation method; Mascot score threshold). New identified peptides are highlighted in grey. (b) The 103 urinary DN type 2 biomarkers established in the PREDICTIONS cohort and their distribution in the DN type 1 cohort of the Rossing study [1]. Shown are the protein identification number in the data set (ID), mass (in Dalton), and normalized CE-migration time (in minutes). Furthermore, p-values of the multivariate statistic of the trainings set are listed (BH = Benjamini-Hochberg; BY = Benjamini-Yekutieli) and p-values of the used U-test for the test set. Significant biomarkers are marked in bold letters.
(0.06 MB XLS)

Table S4 Rank correlation of the 65 previously defined urinary DN biomarkers [1]. Shown are the protein identification numbers (ID), the correlation with urinary albumin excretion (UAE) and with creatinine clearance (CrCl), respectively. The results of the correlation analysis include the significance level P and the Spearmann's coefficient rho. In the last column SwissProt names of the sequenced peptides are shown. All italic marked values indicate no significance.
(0.02 MB XLS)
