# Supplementary material for: Multicentric Validation of Proteomic Biomarkers in Urine Specific for Diabetic Nephropathy
Source: PLoS One. 2010 Oct 20;5(10):e13421. doi: 10.1371/journal.pone.0013421 (PMC2958112; doi:10.1371/journal.pone.0013421)
Supplement: Figure S1 — The mass spectra annotated with fragment assignments of all identified peptides (see Protein ID) from the Mascot searches are shown. (0.28 MB PPT) [file pone.0013421.s001.ppt]

## Slide 1
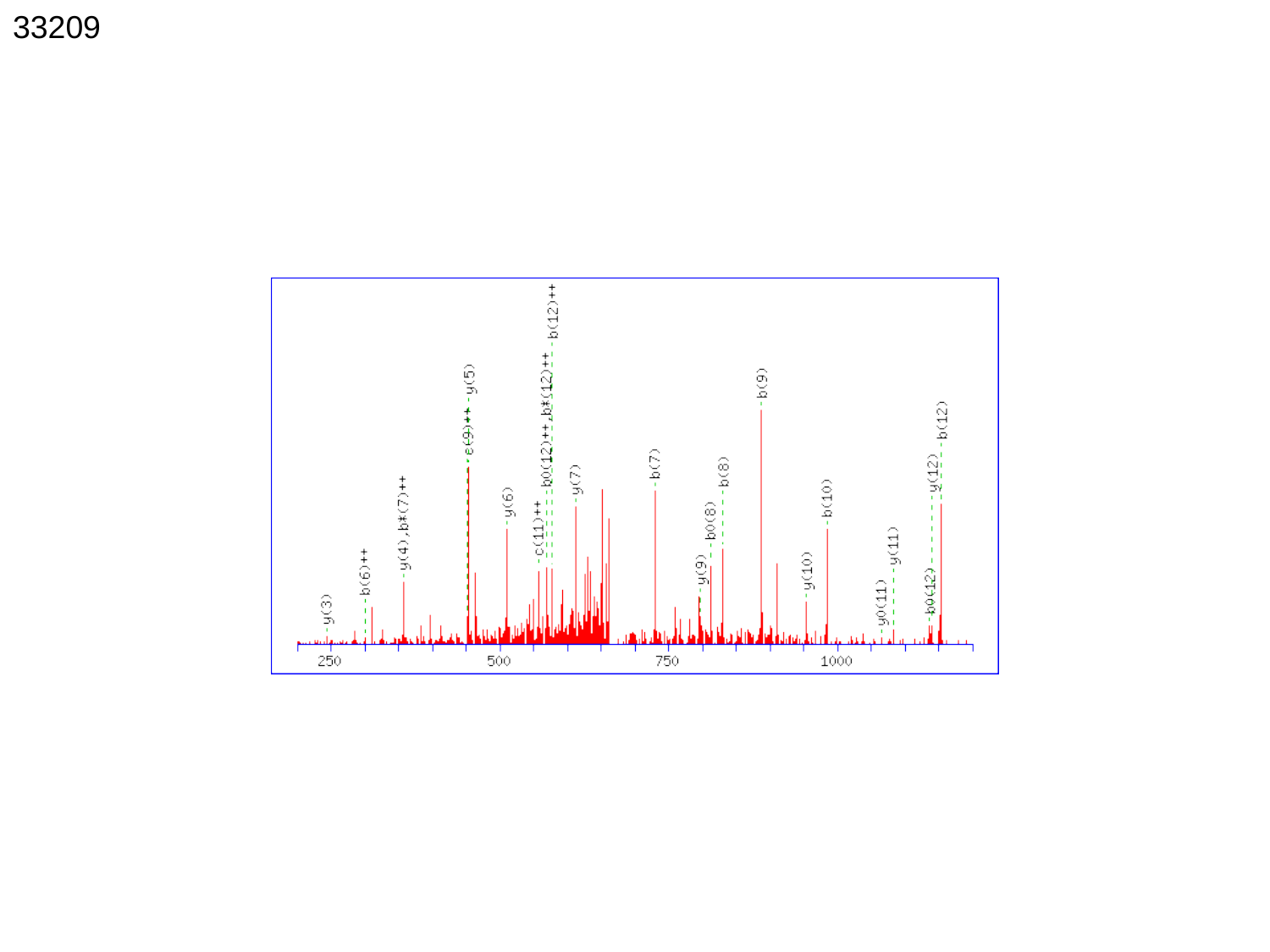

33209

## Slide 2
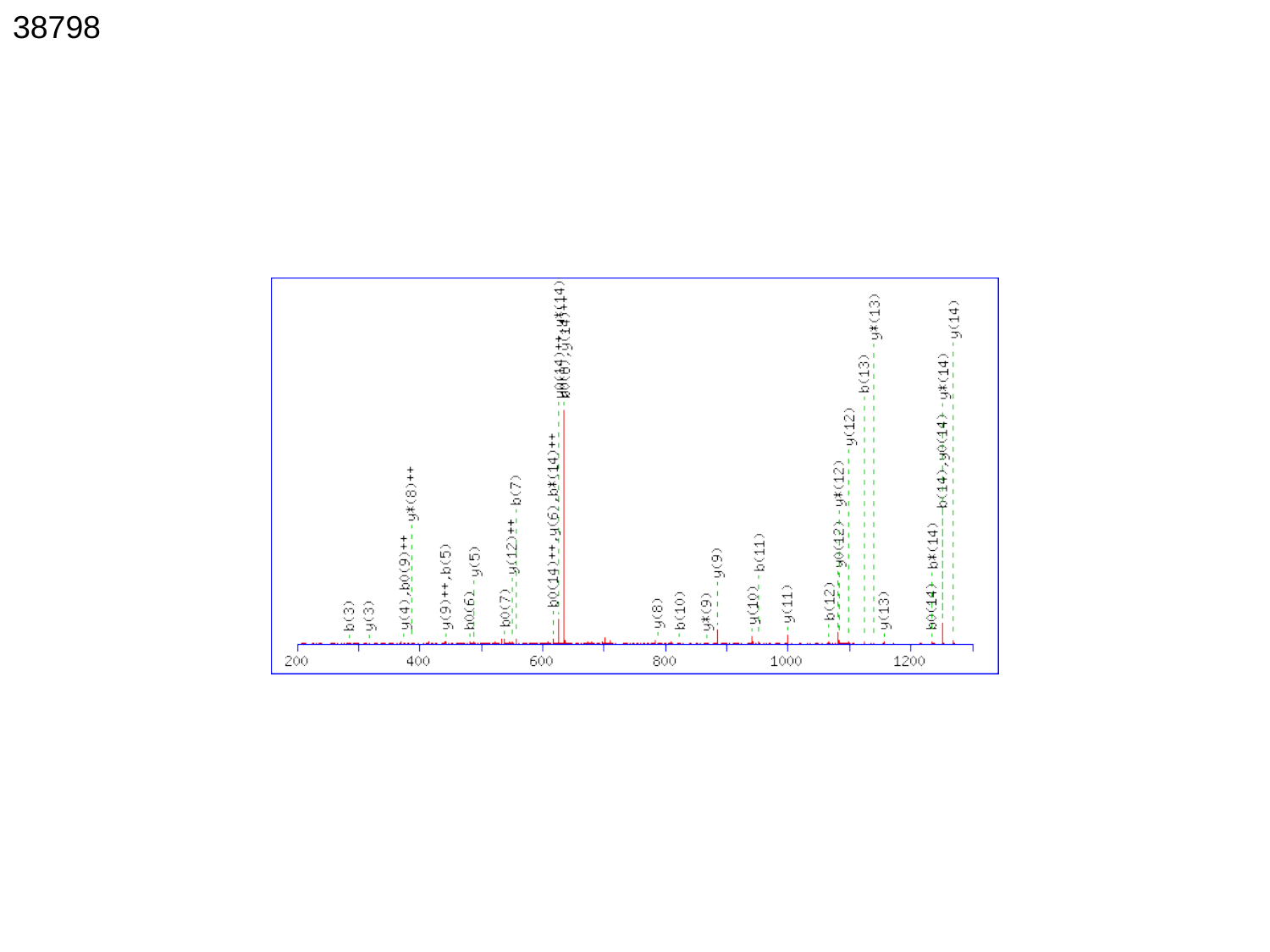

38798

## Slide 3
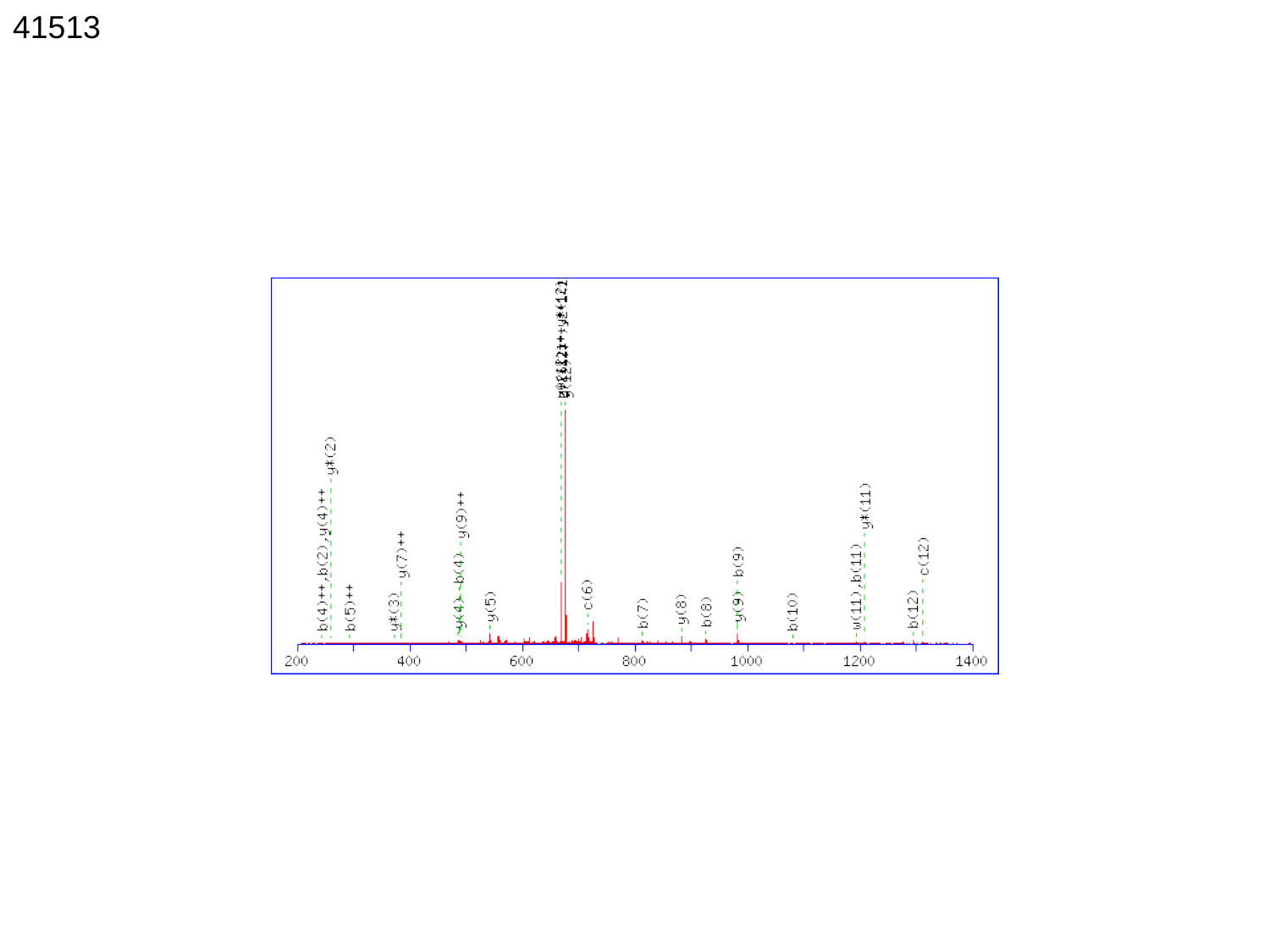

41513

## Slide 4
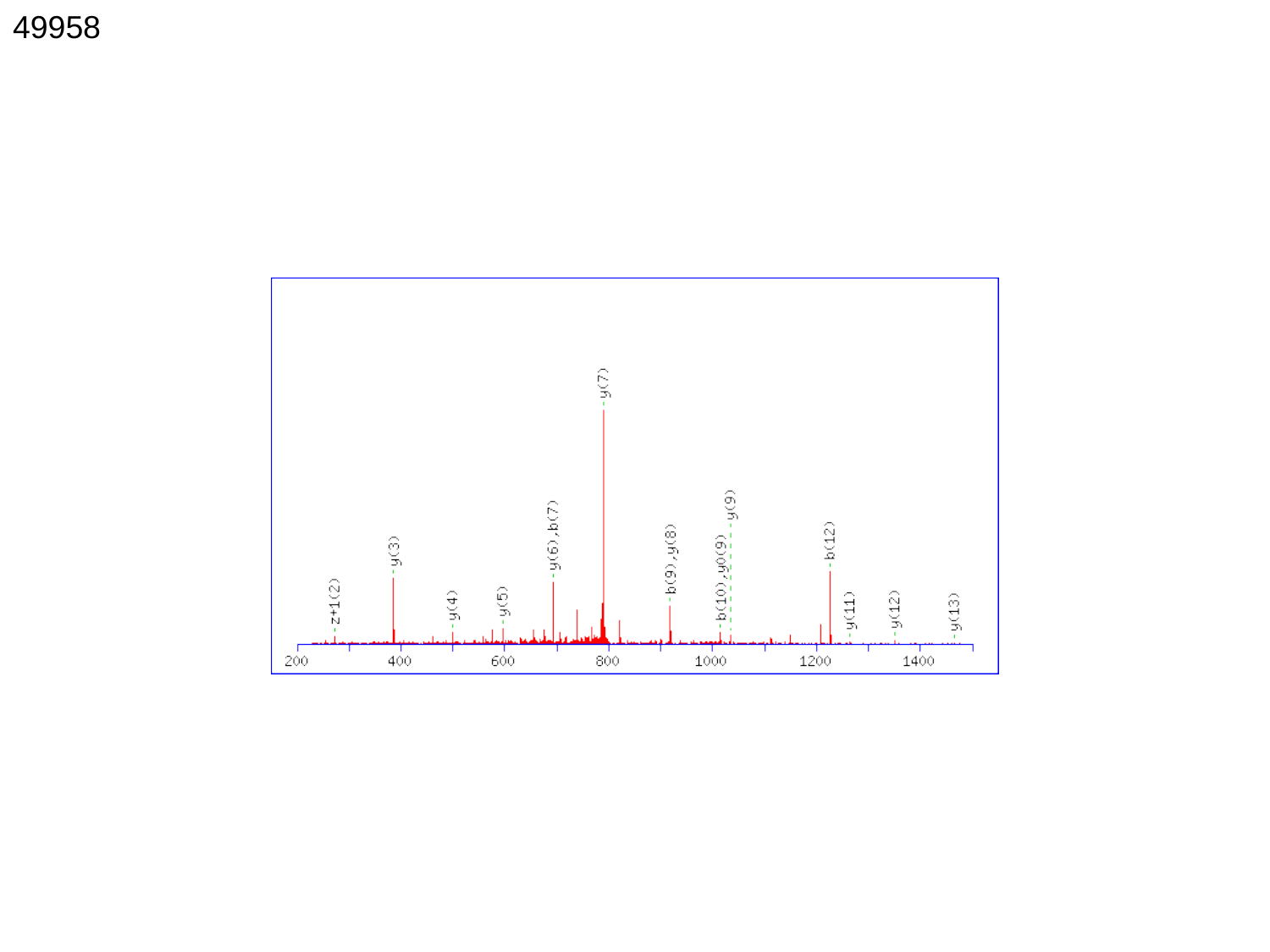

49958

## Slide 5
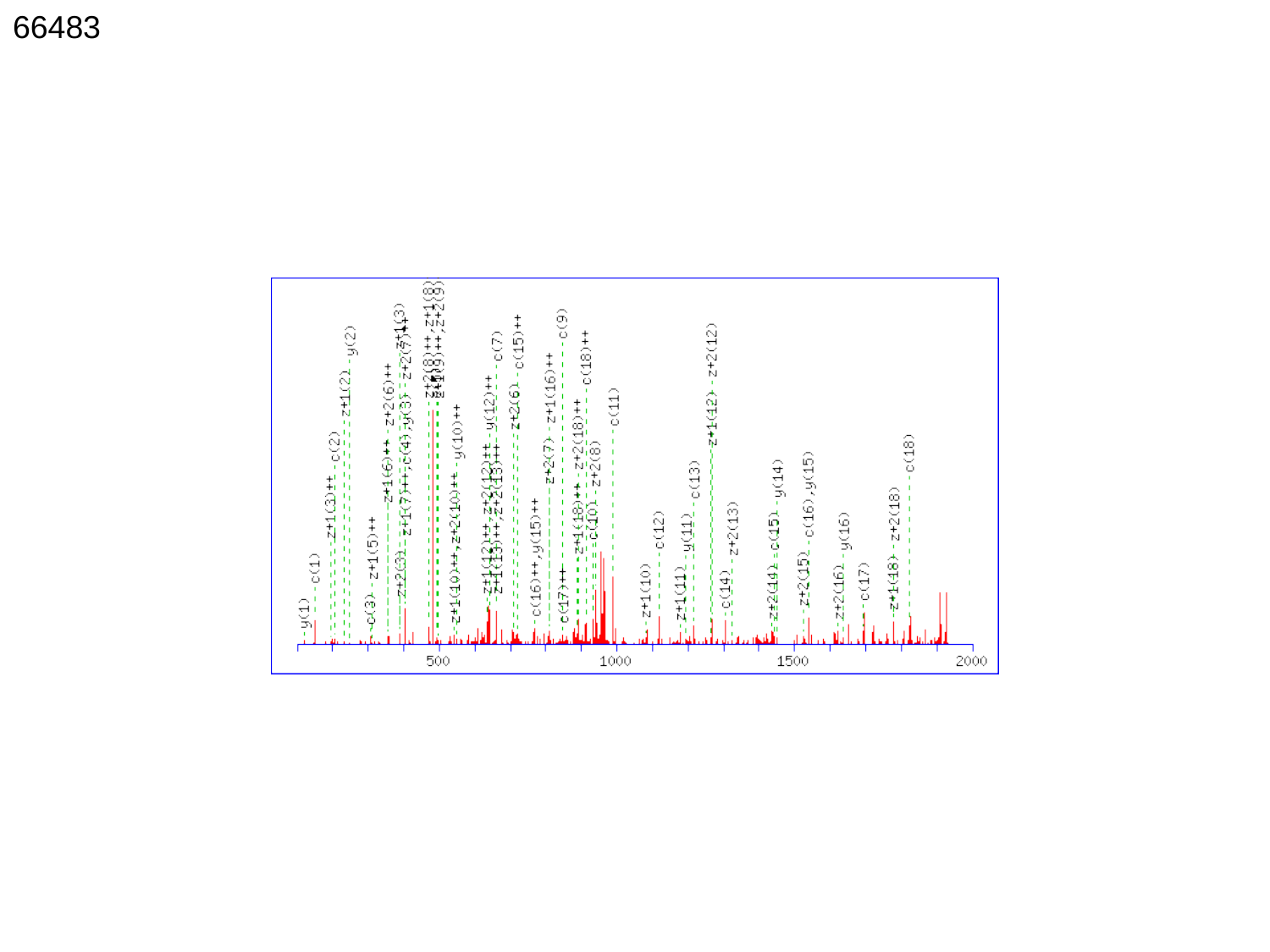

66483

## Slide 6
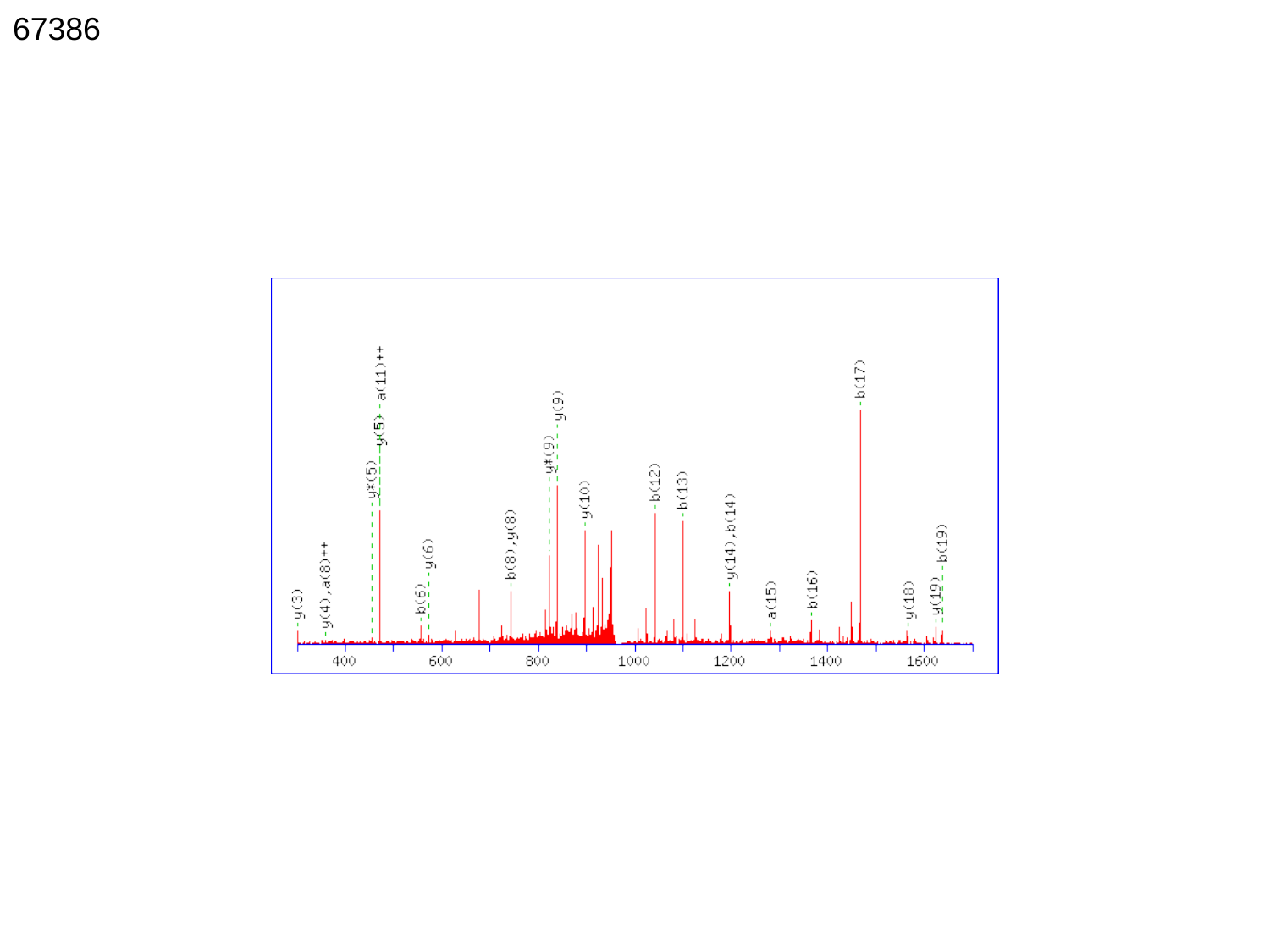

67386

## Slide 7
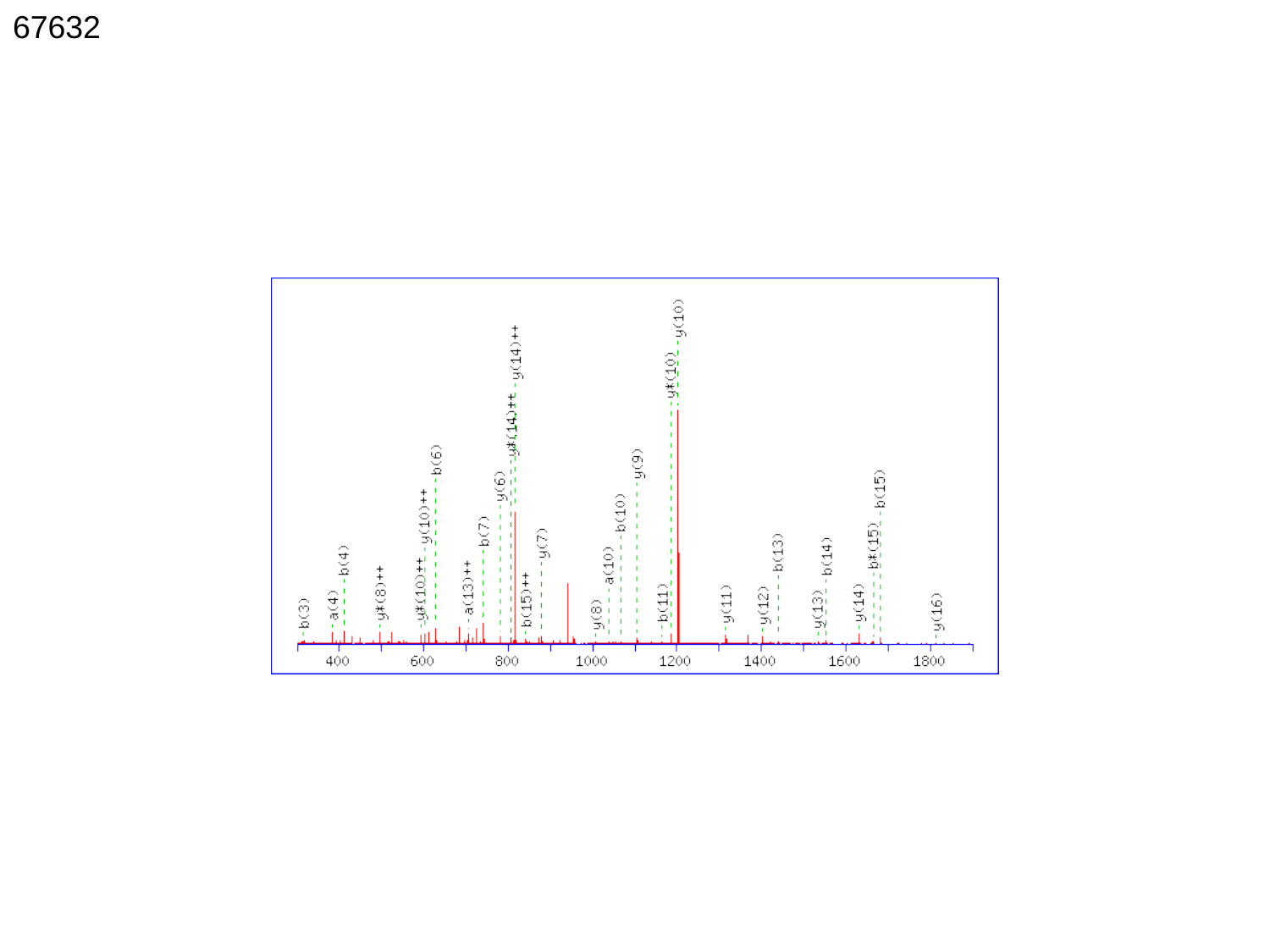

67632

## Slide 8
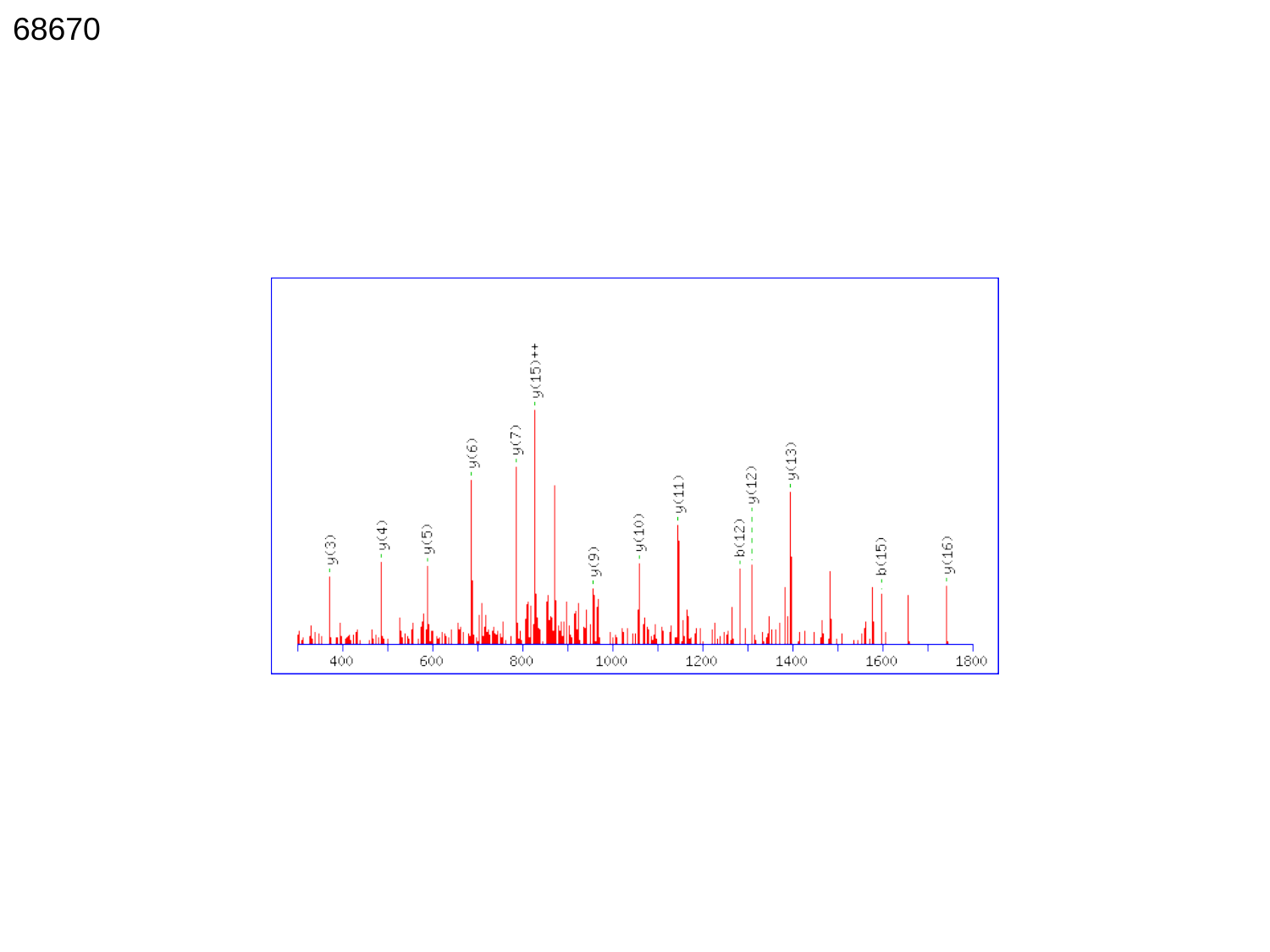

68670

## Slide 9
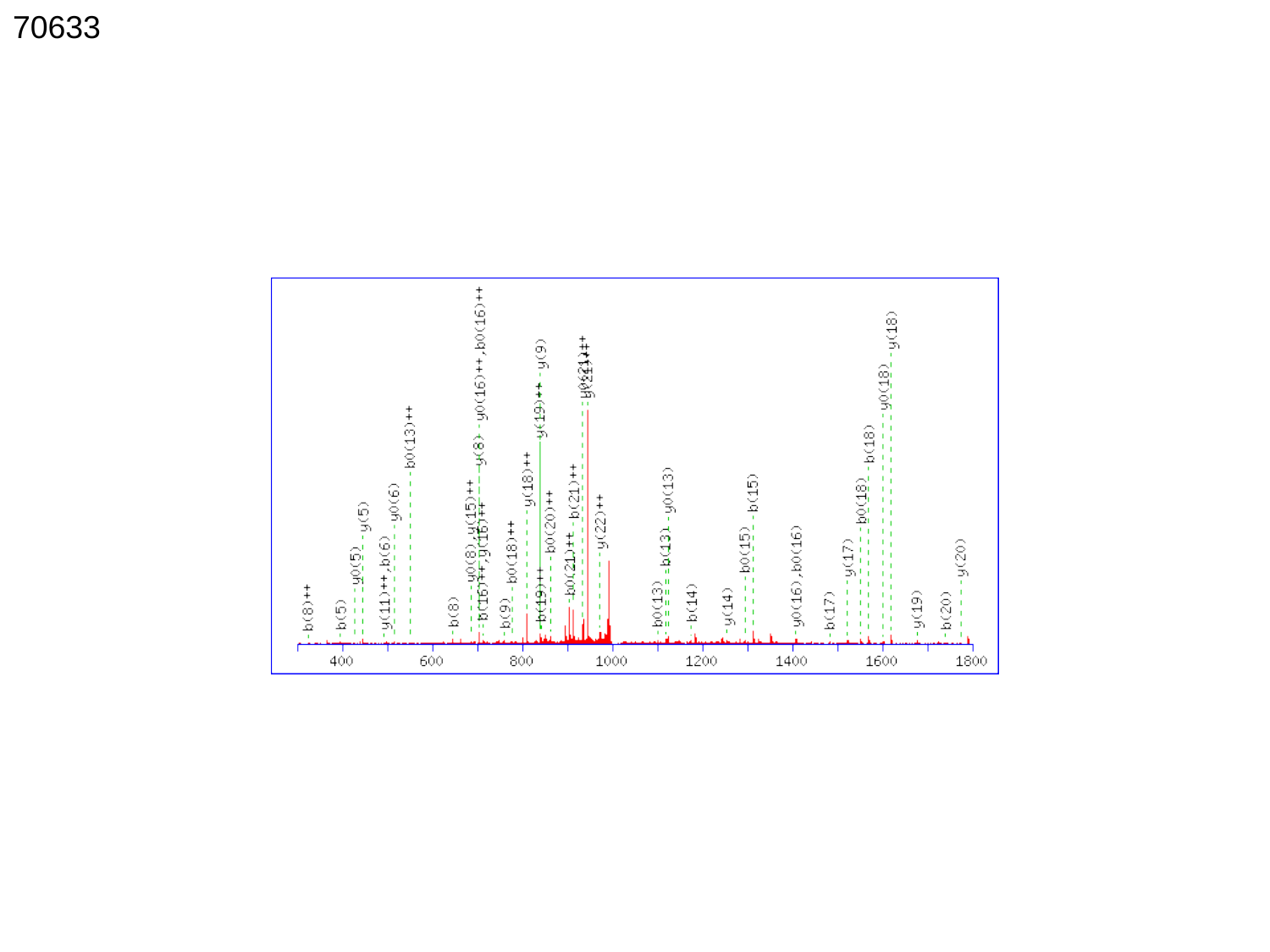

70633

## Slide 10
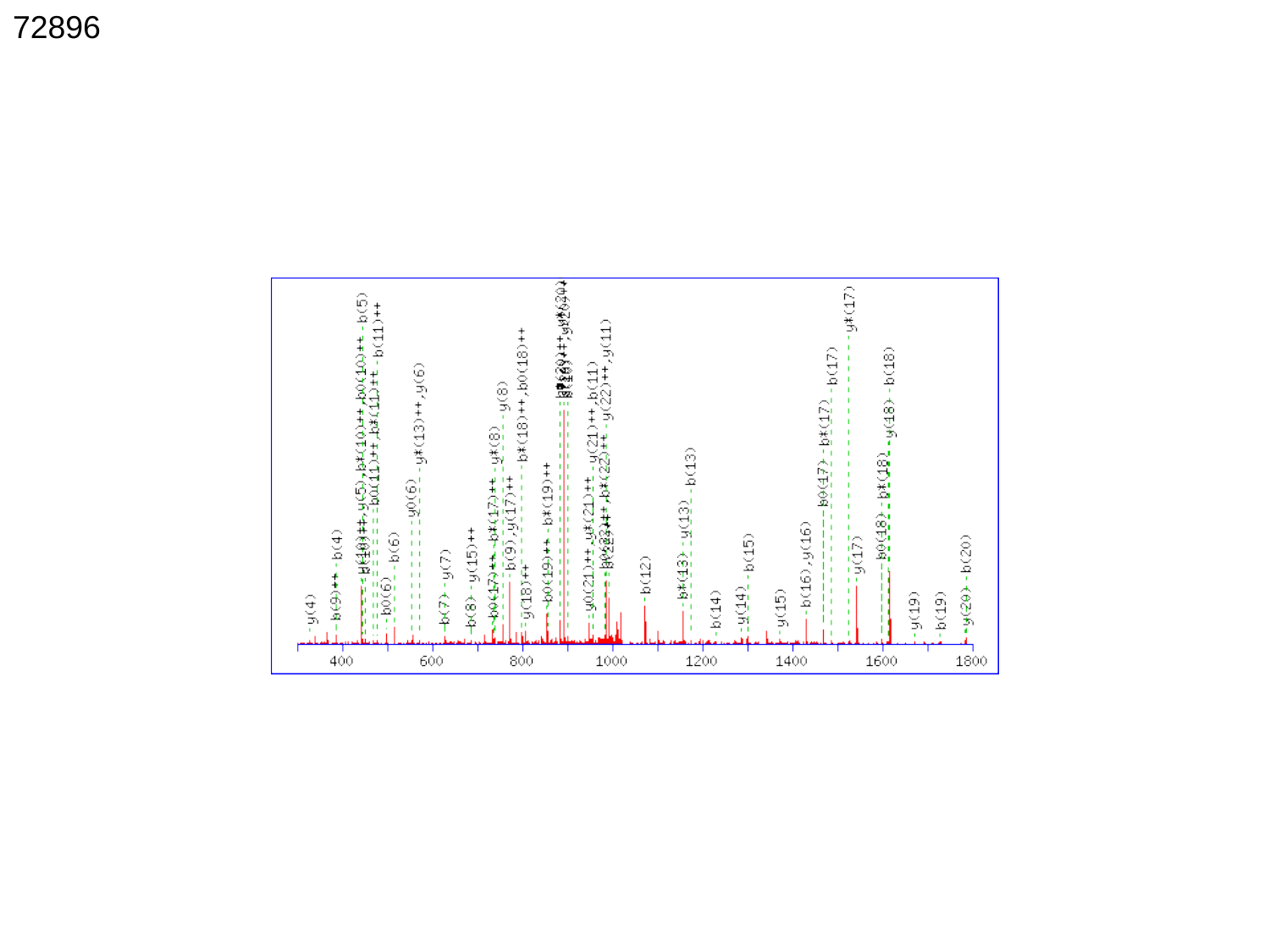

72896

## Slide 11
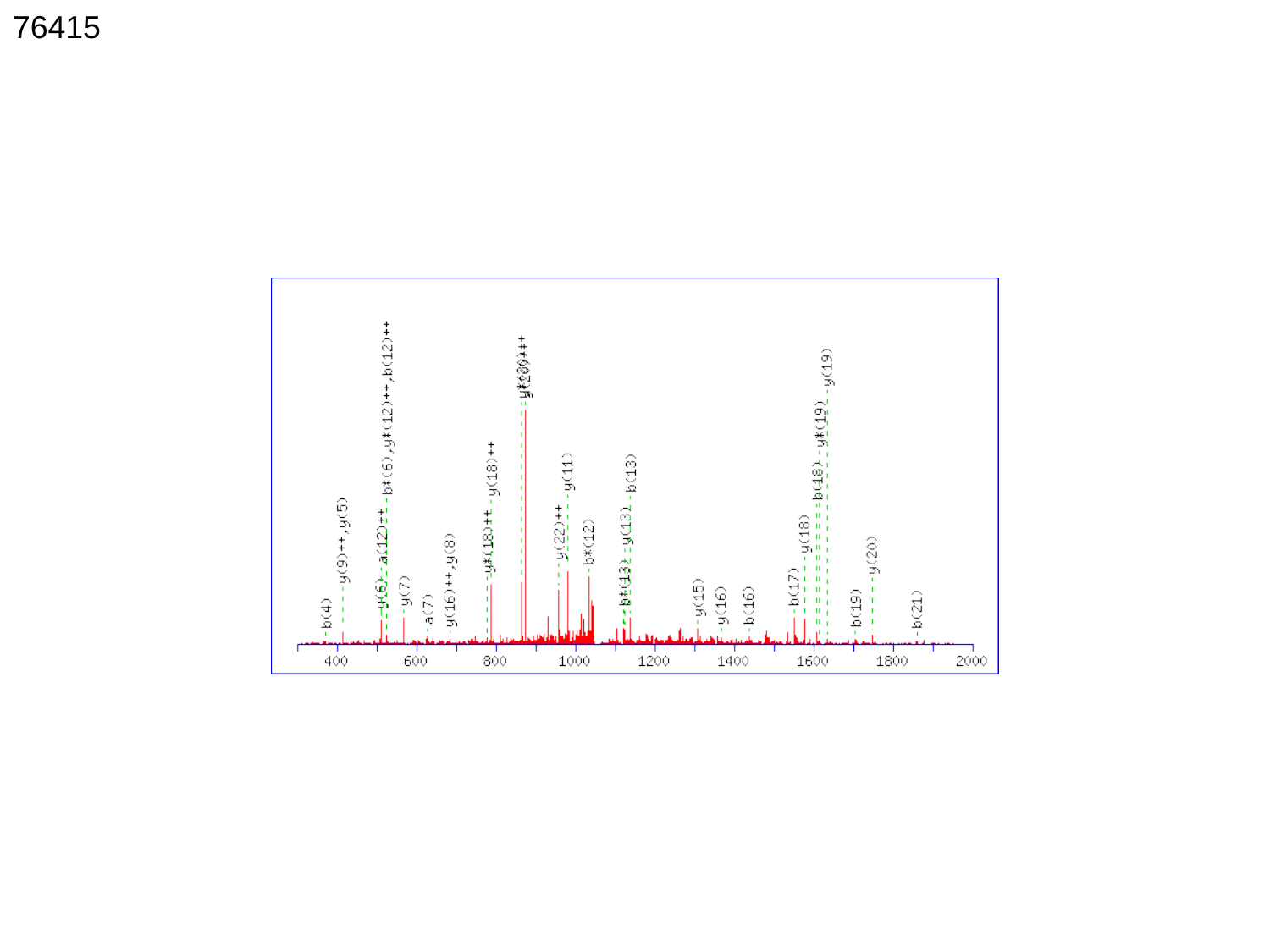

76415

## Slide 12
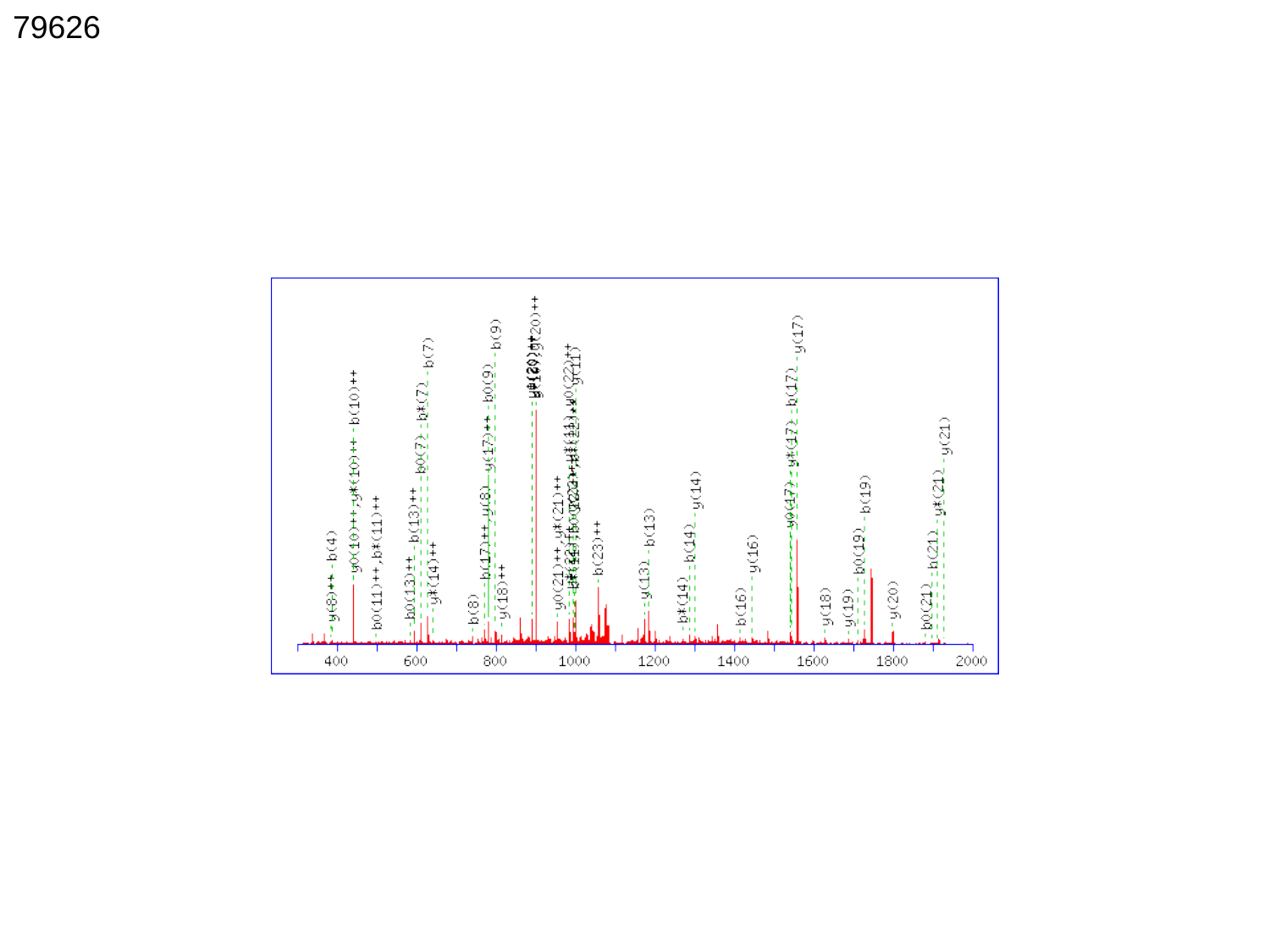

79626

## Slide 13
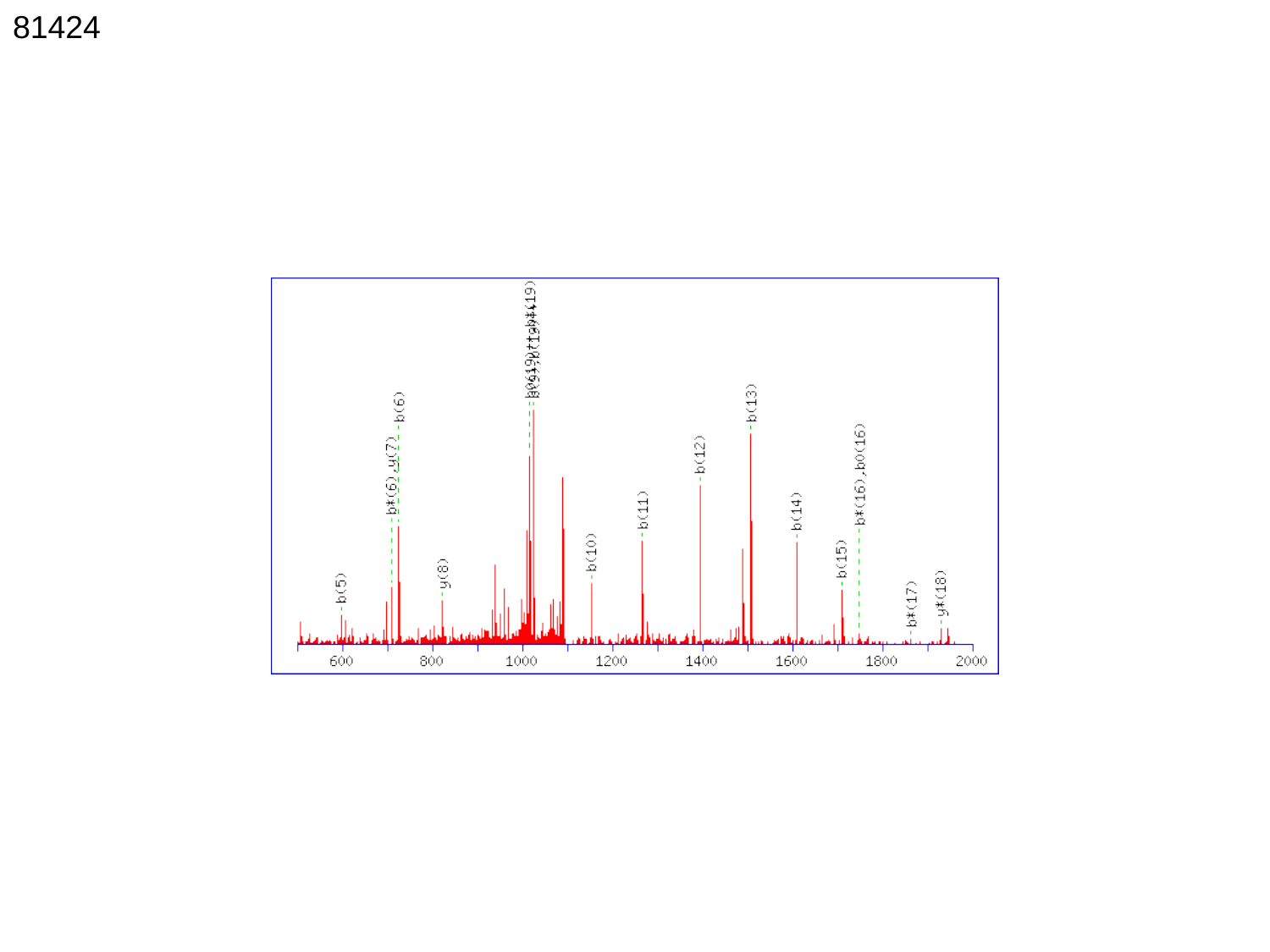

81424

## Slide 14
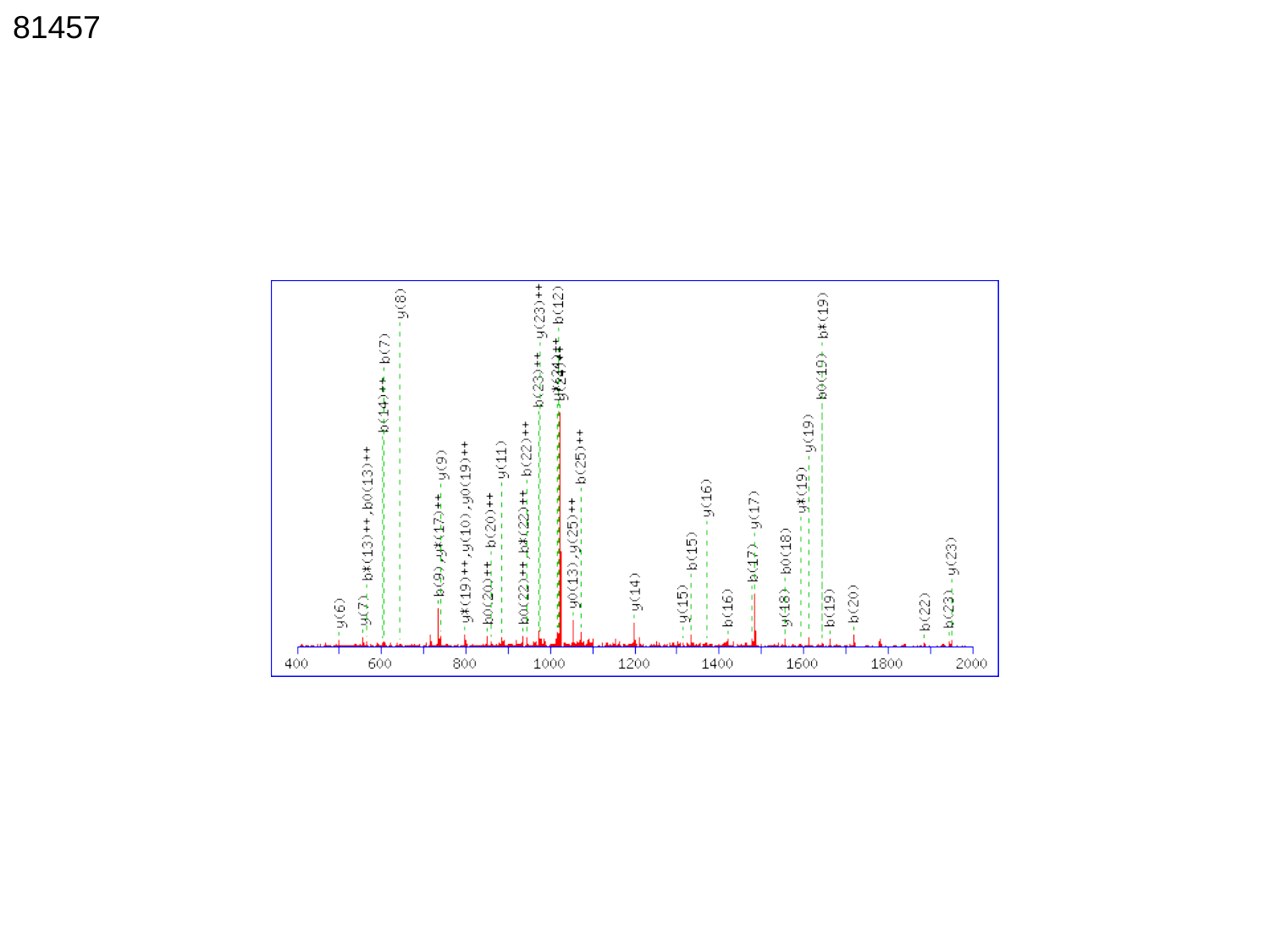

81457

## Slide 15
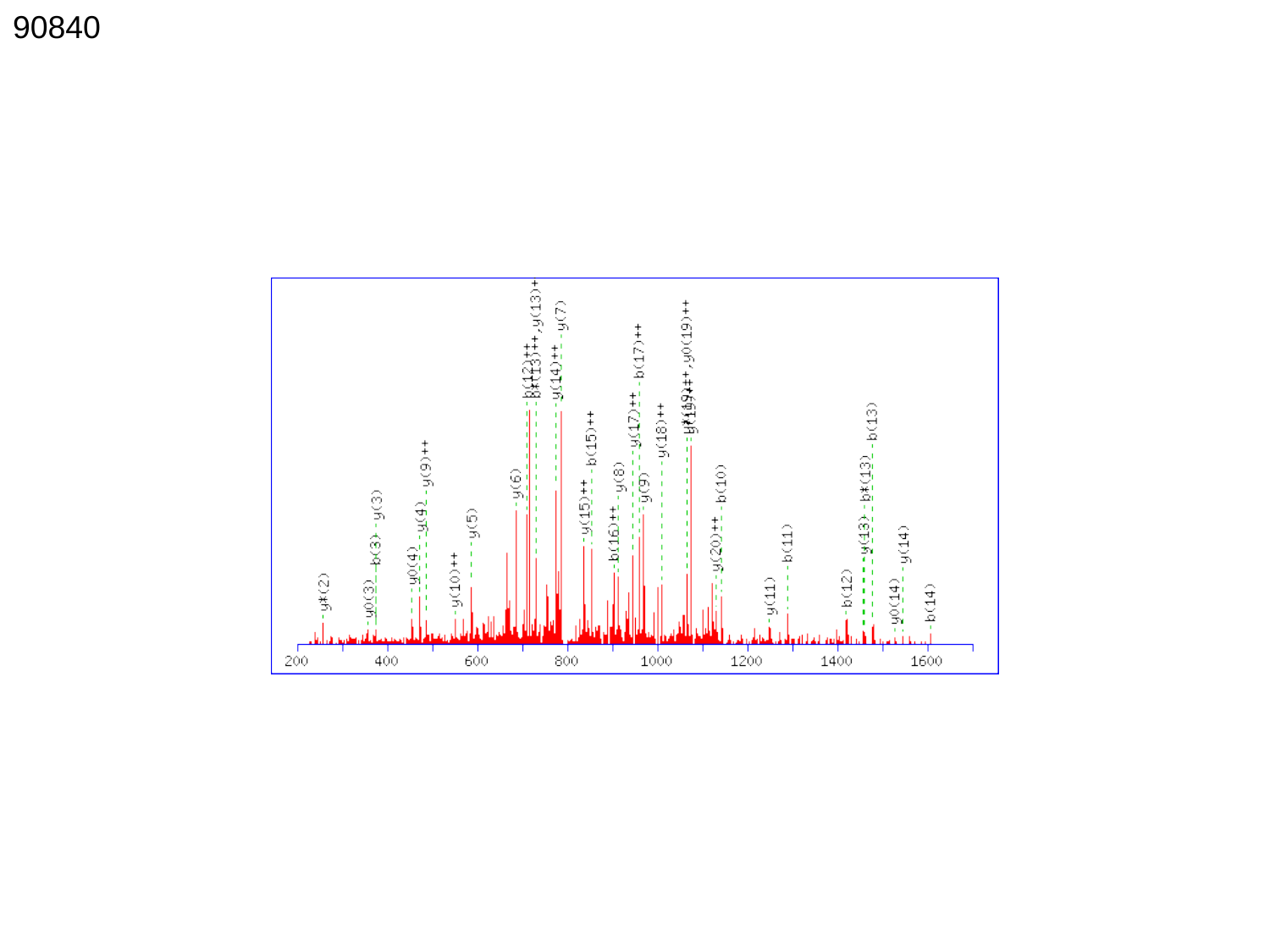

90840

## Slide 16
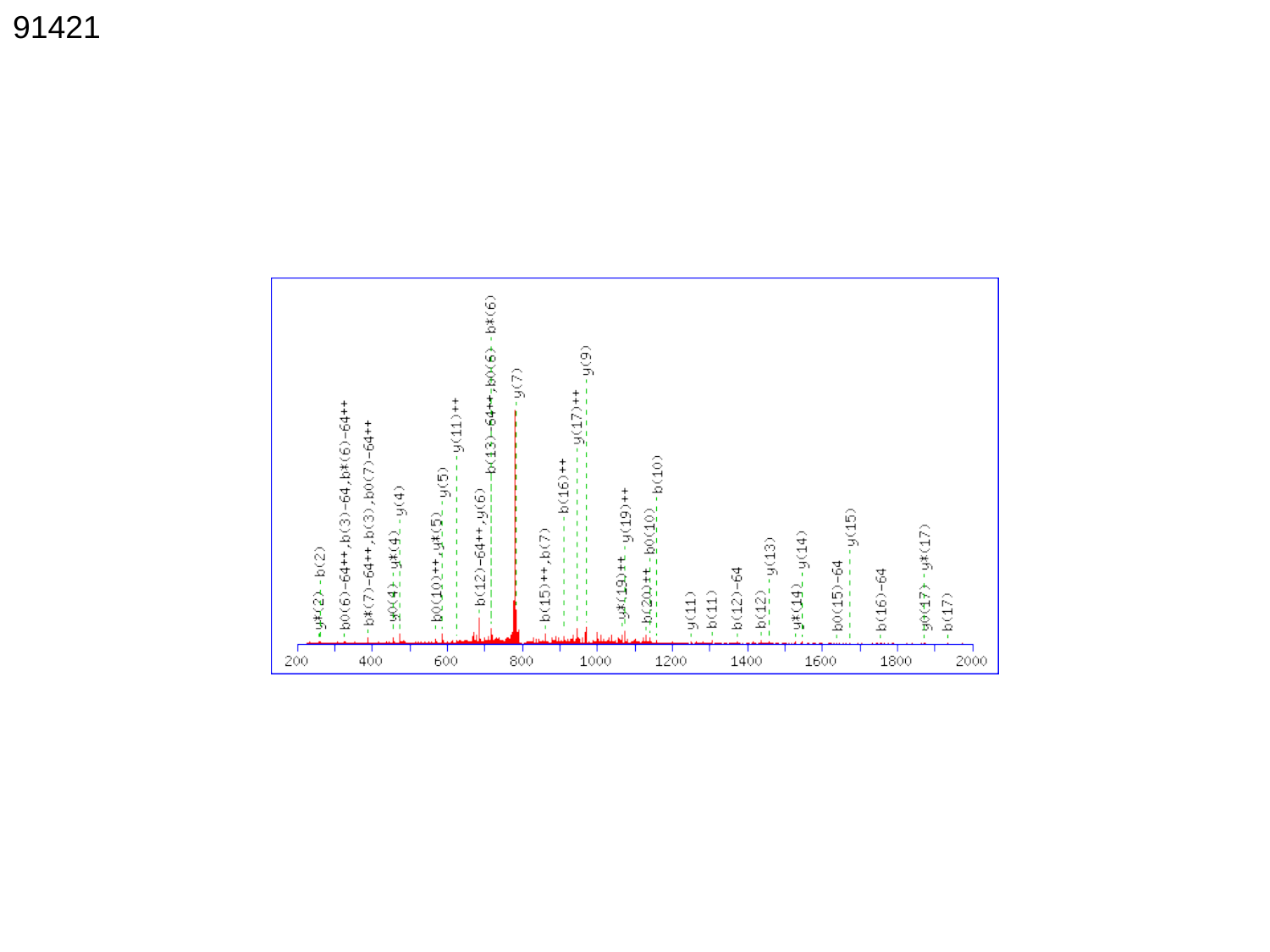

91421

## Slide 17
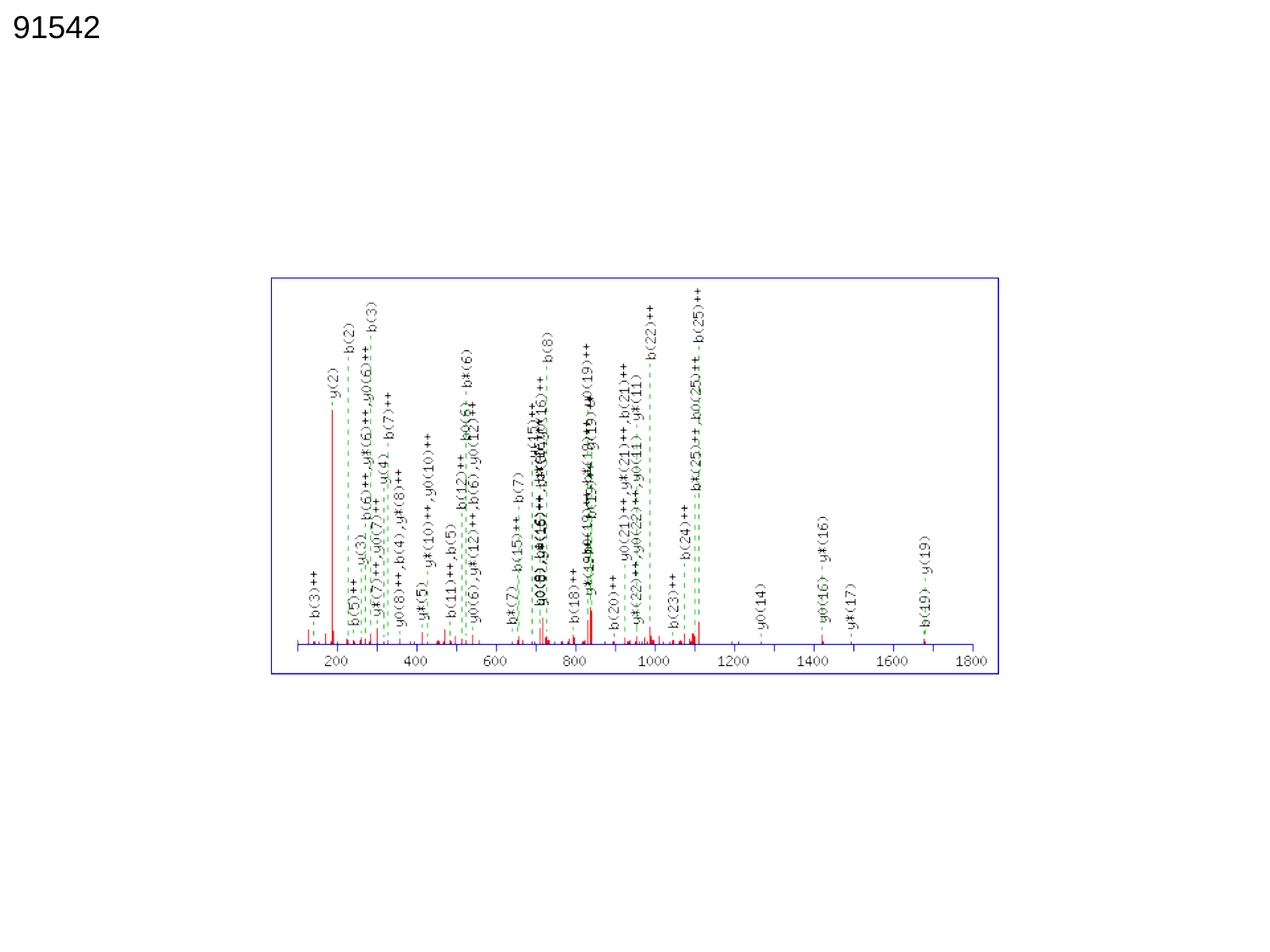

91542

## Slide 18
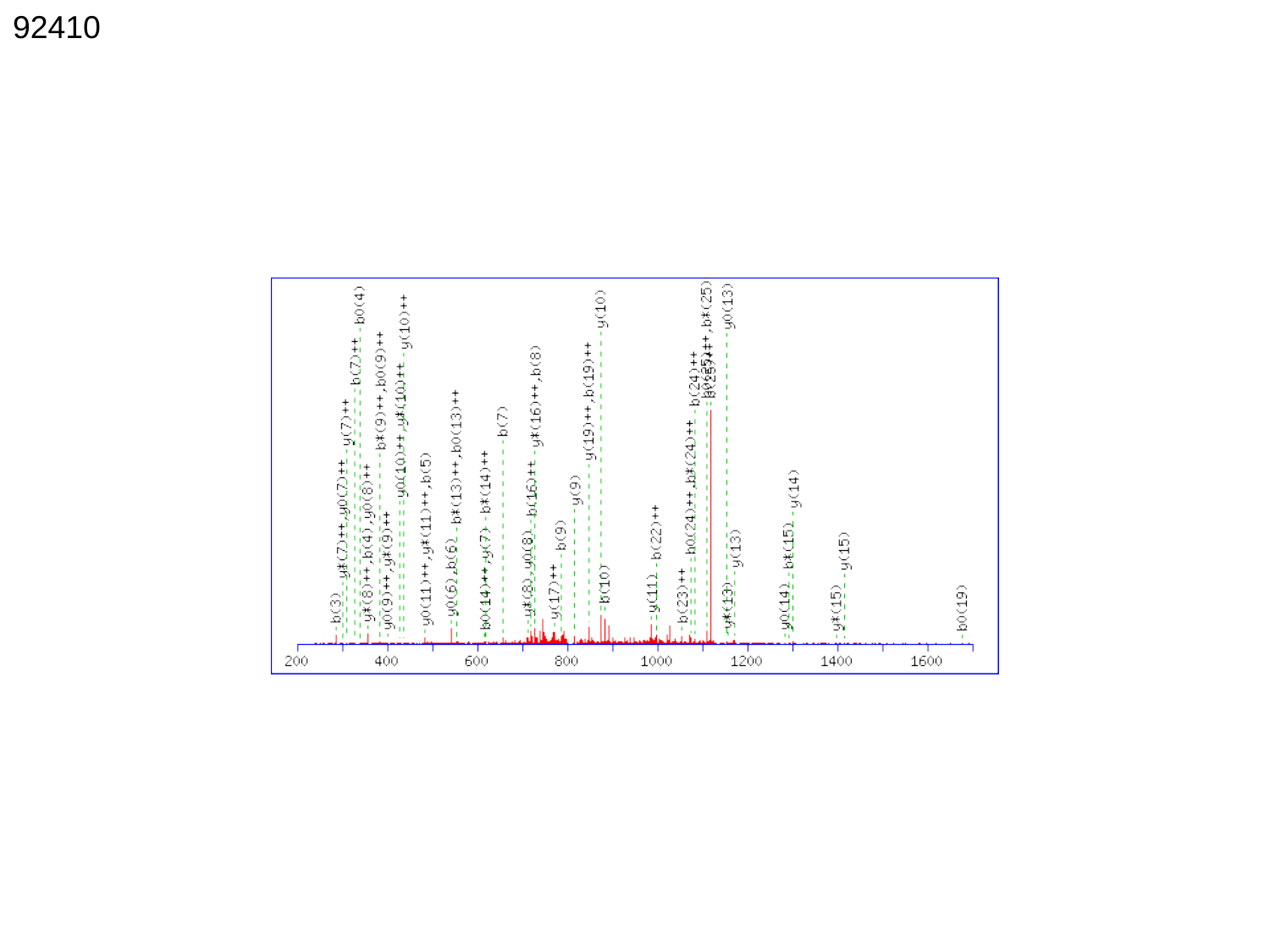

92410

## Slide 19
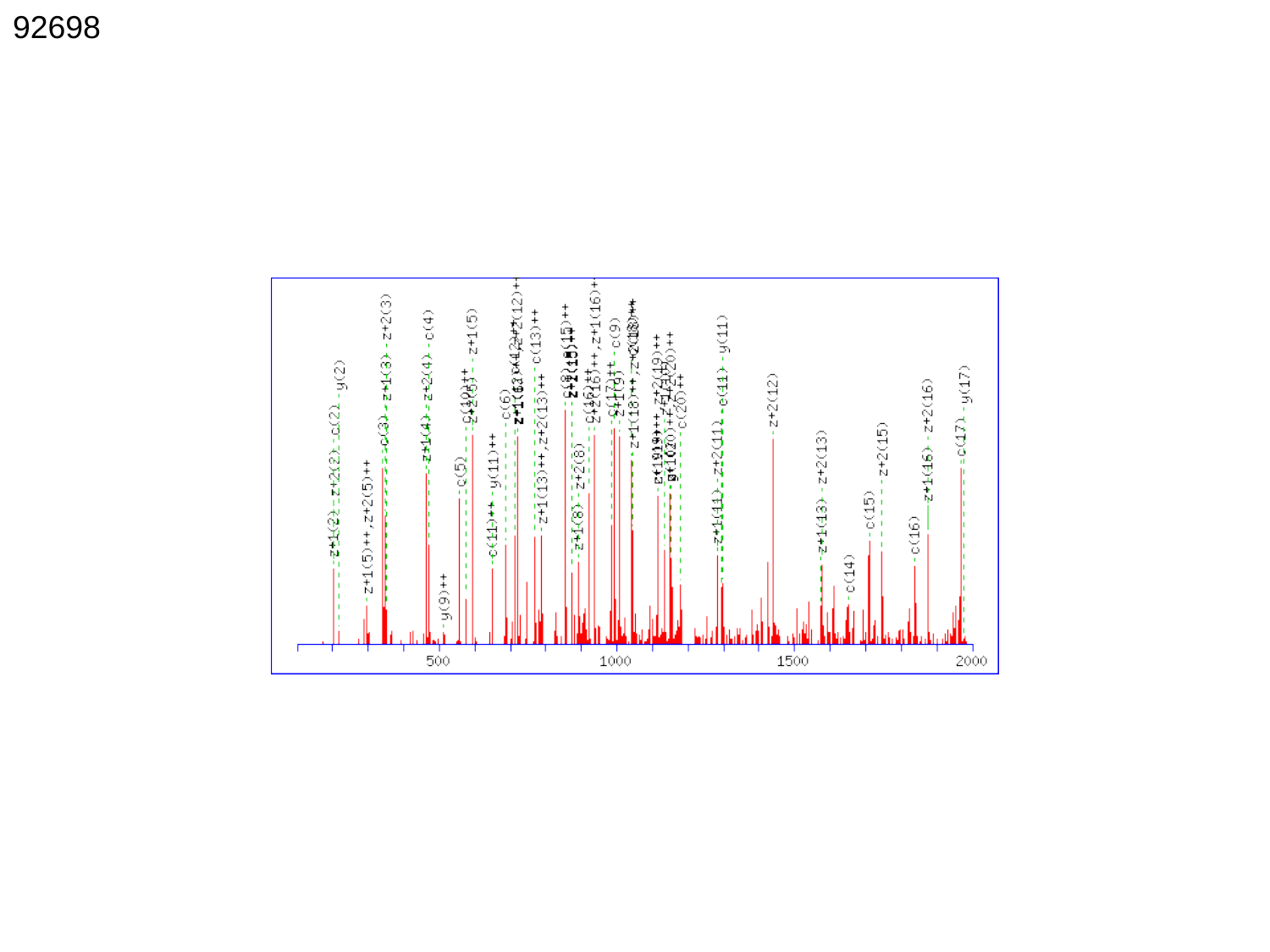

92698

## Slide 20
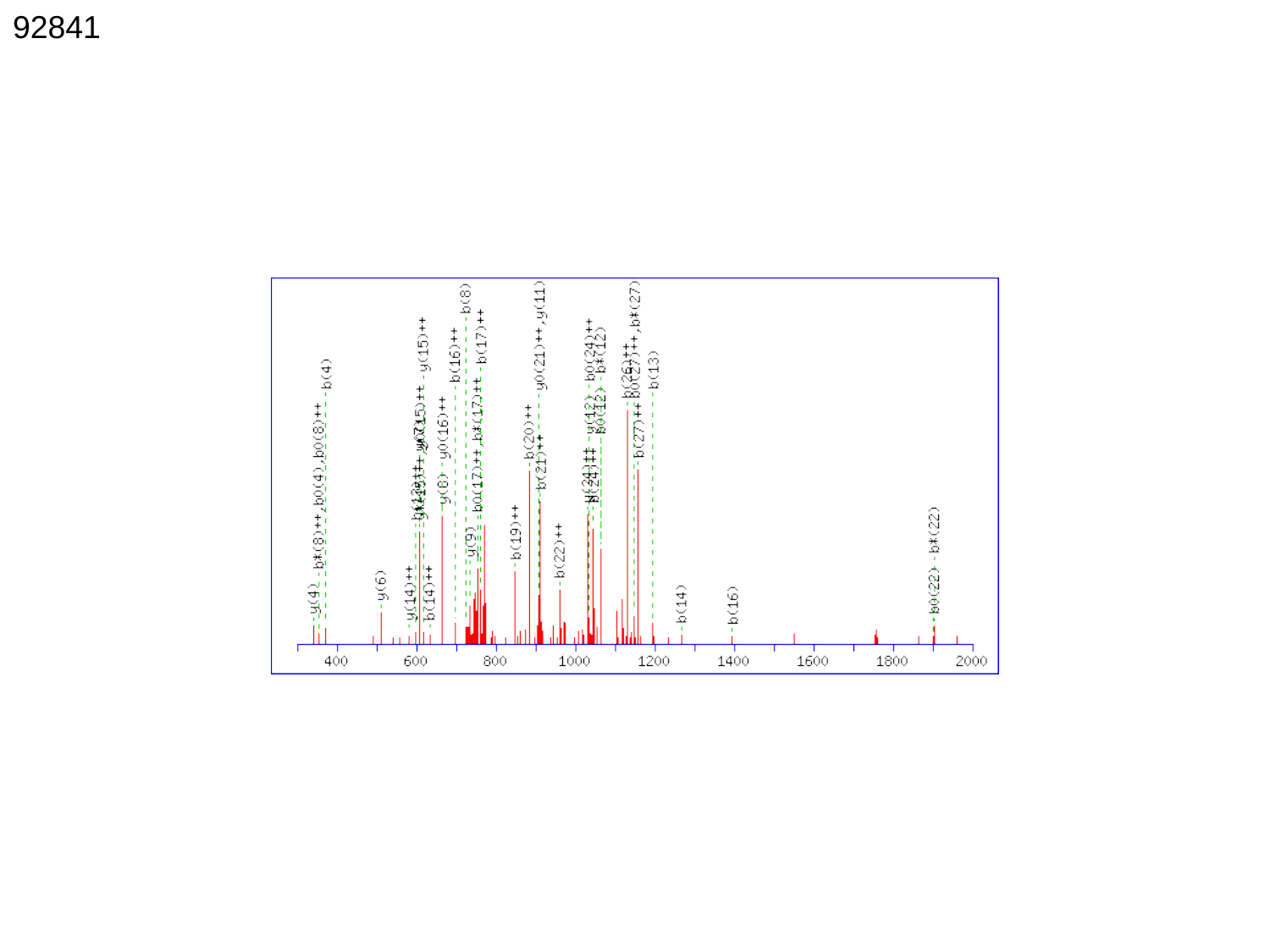

92841

## Slide 21
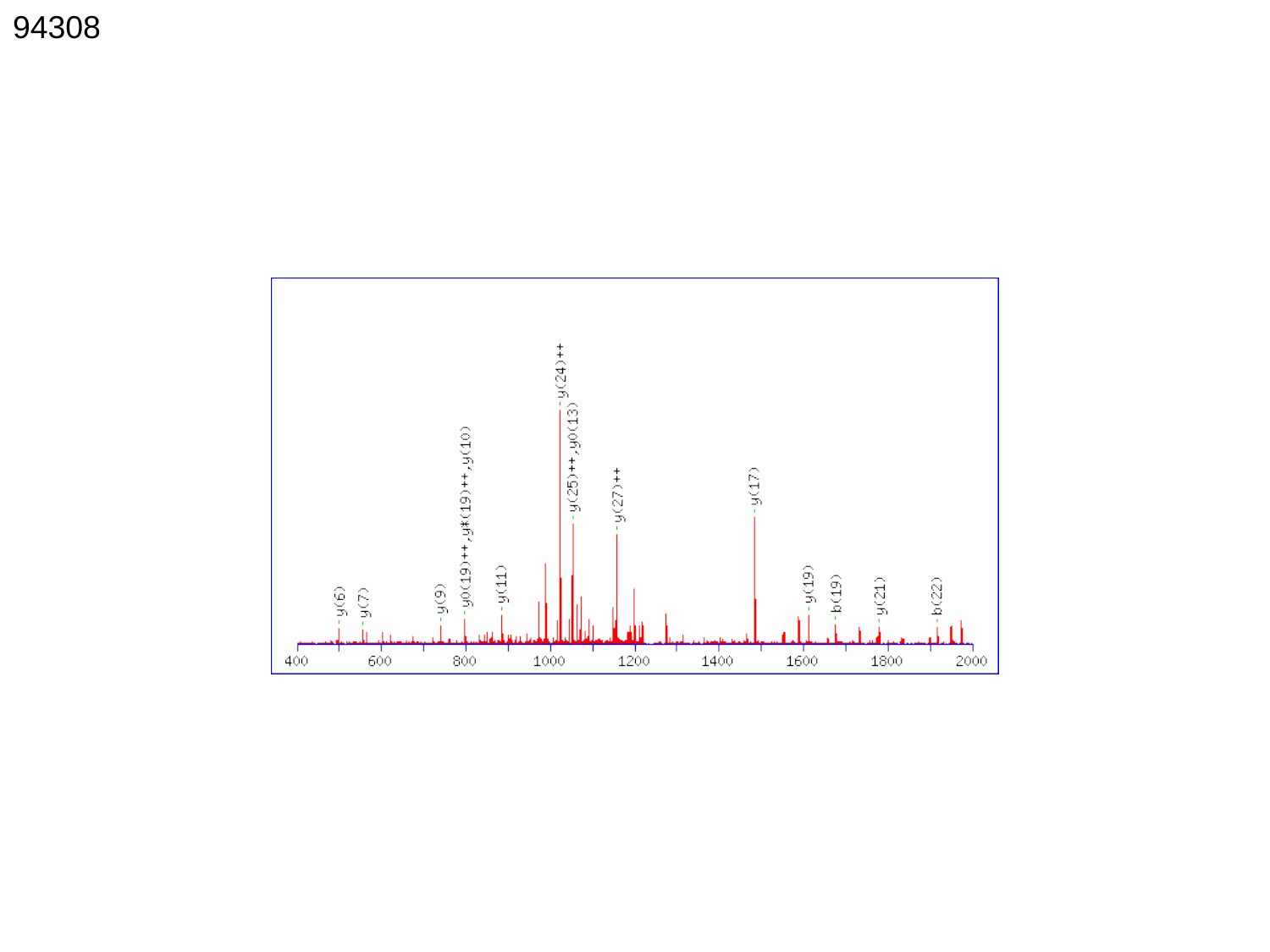

94308

## Slide 22
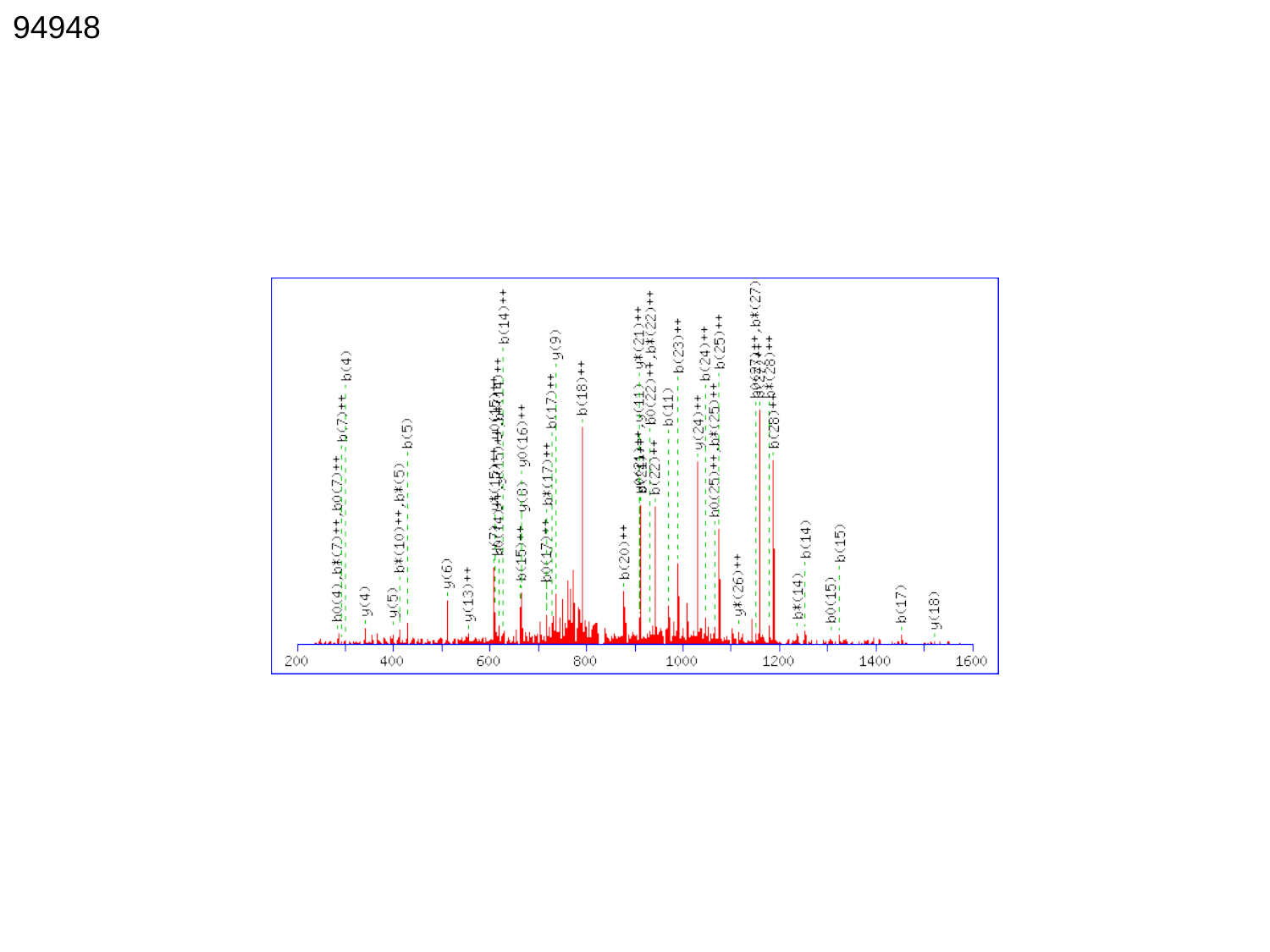

94948

## Slide 23
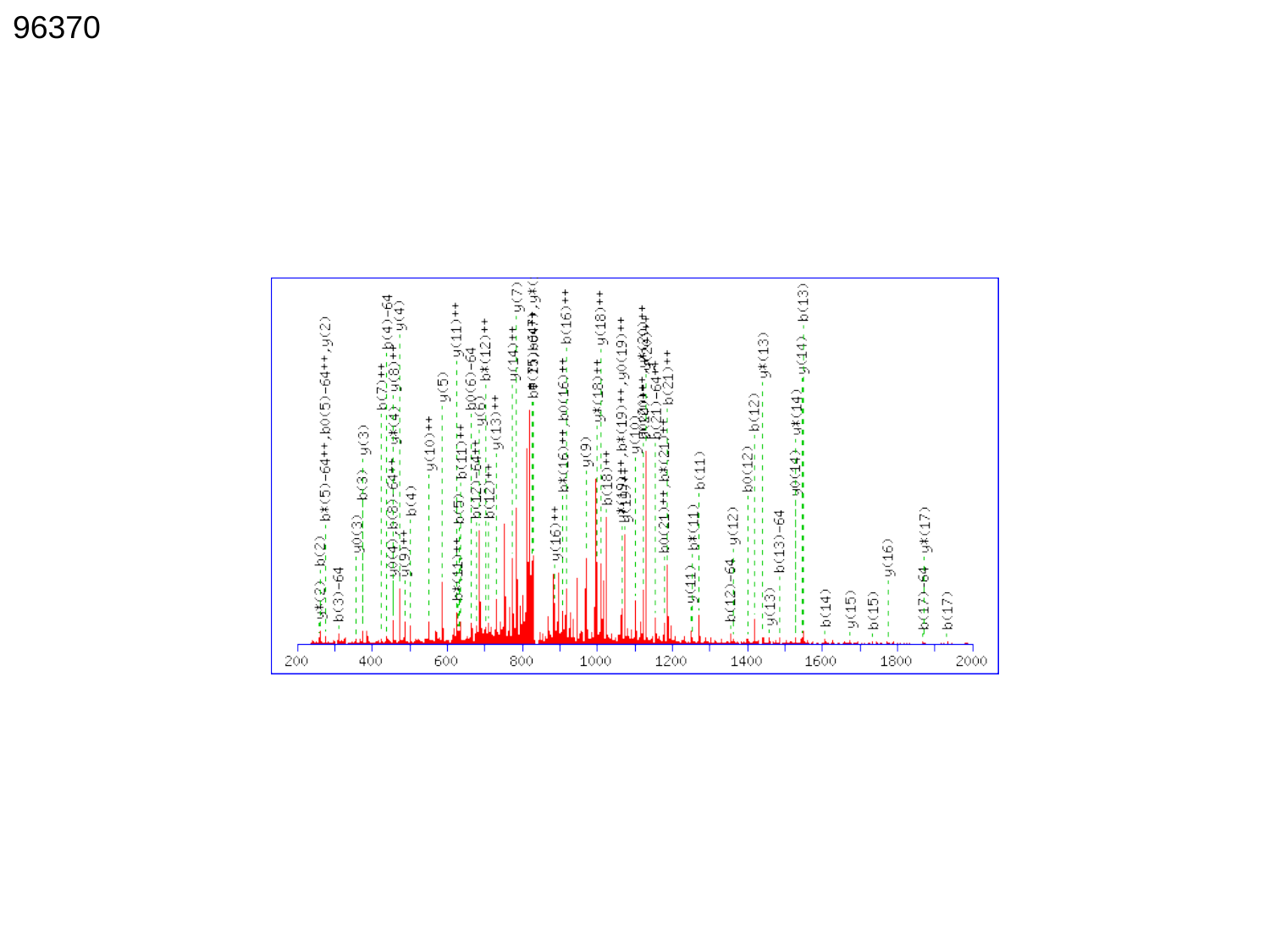

96370

## Slide 24
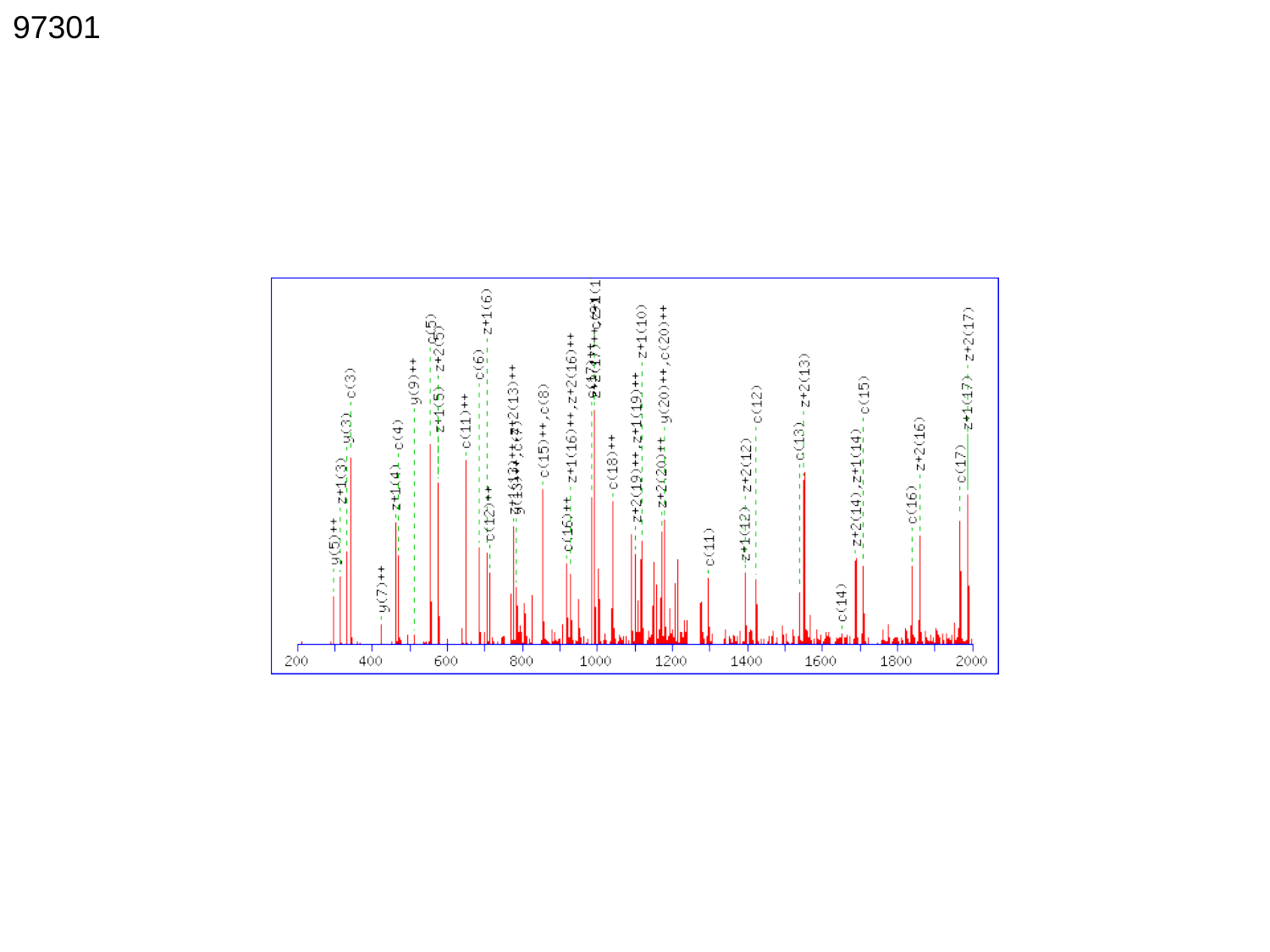

97301

## Slide 25
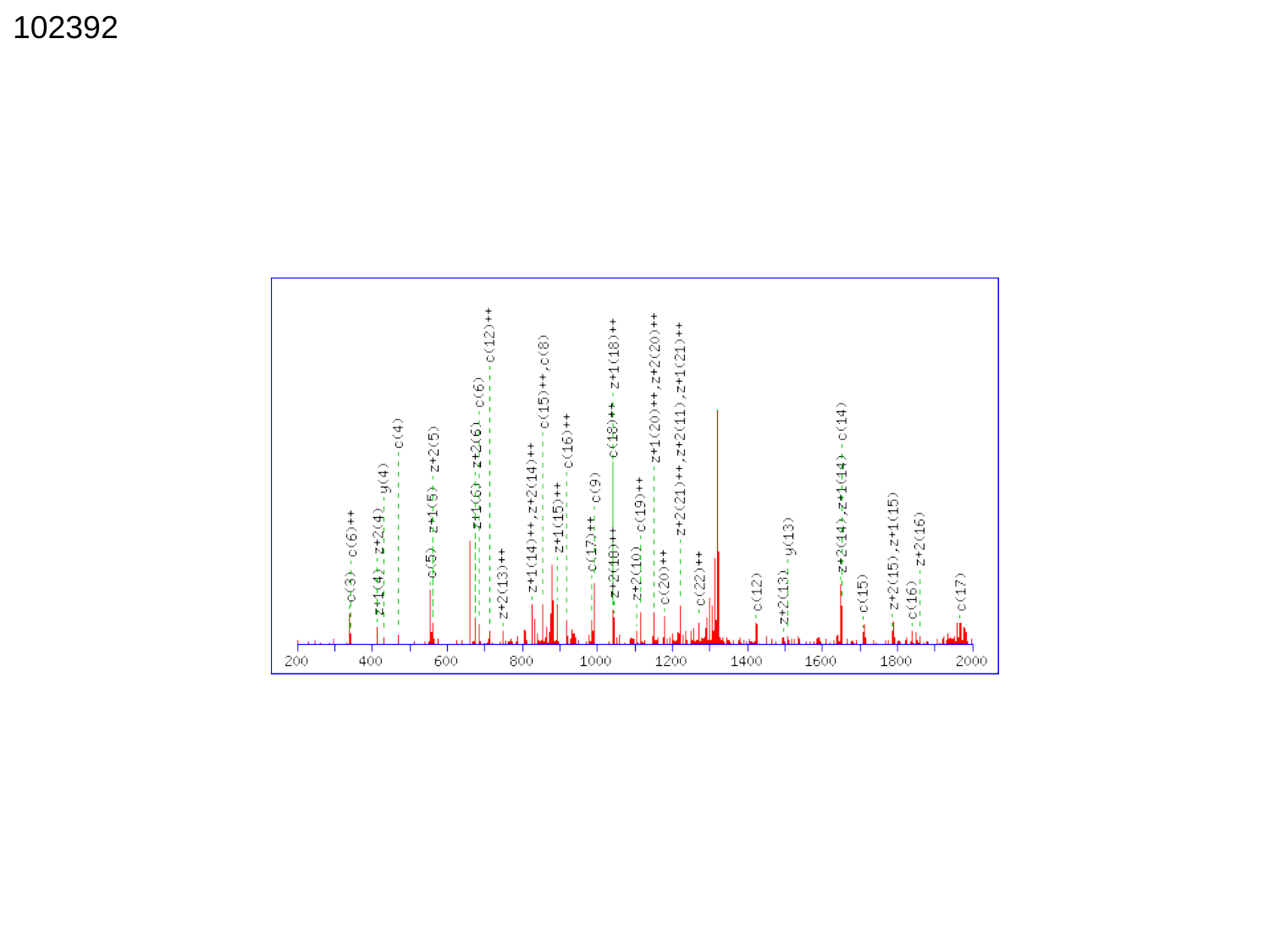

102392

## Slide 26
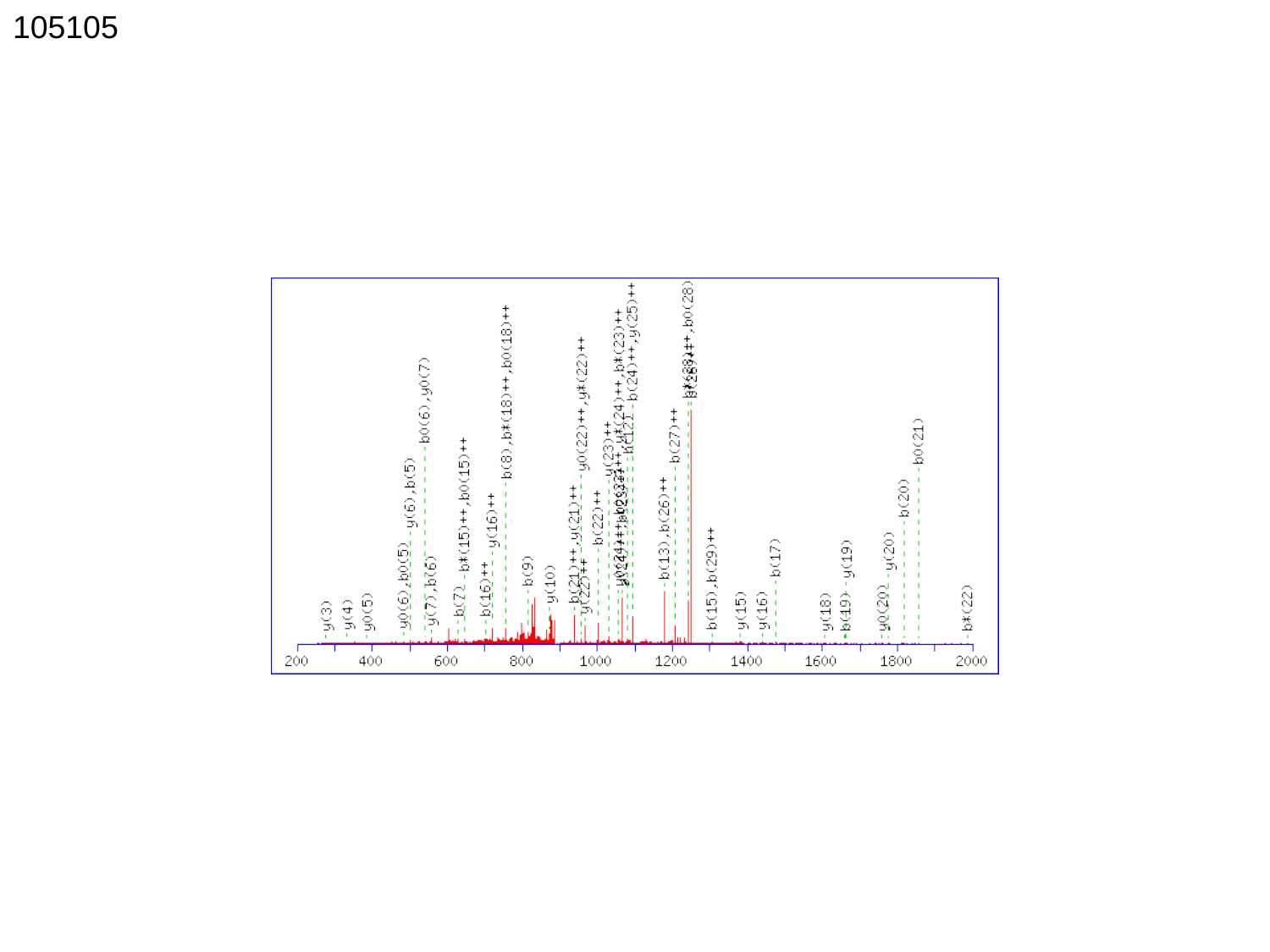

105105

## Slide 27
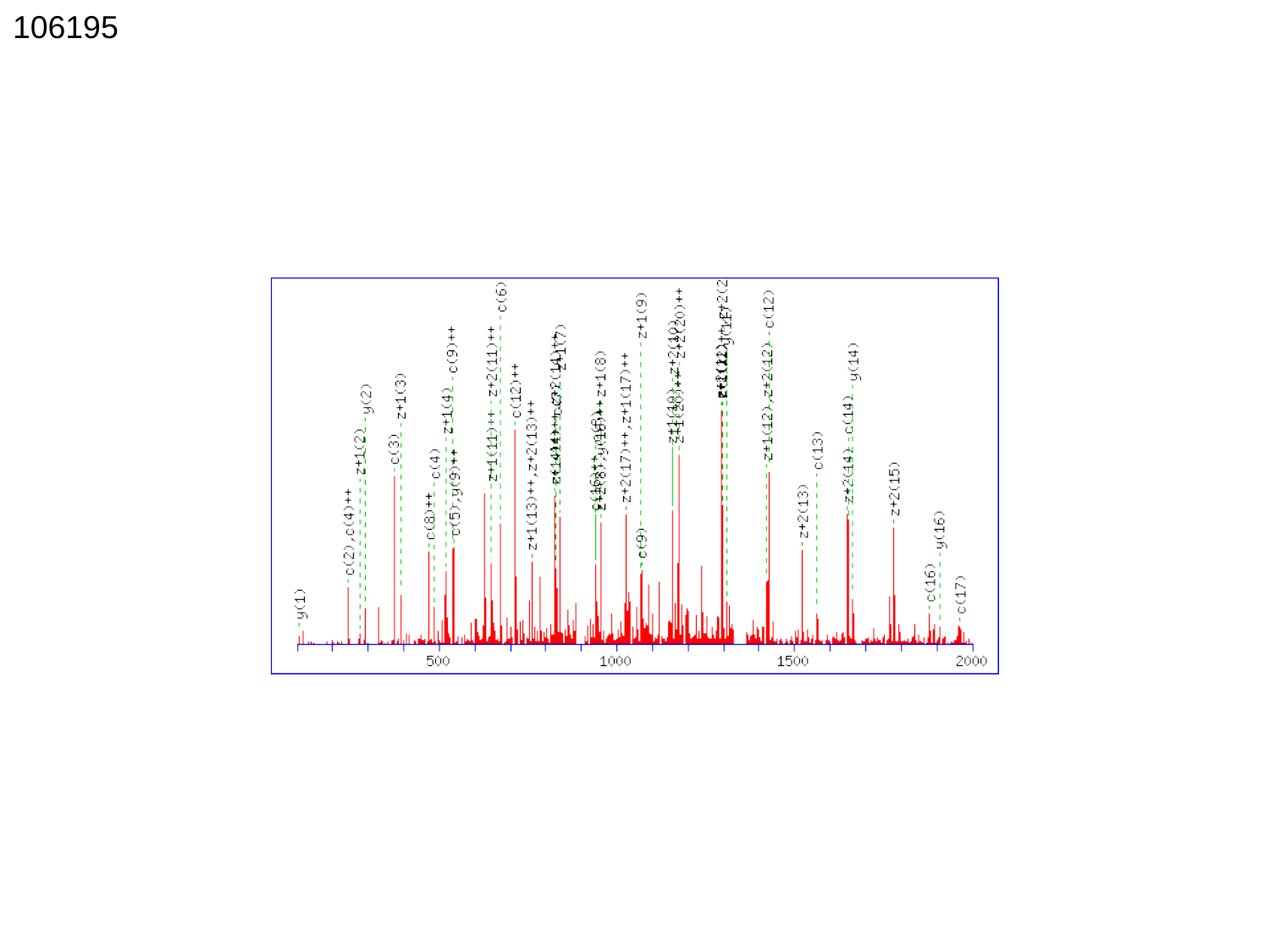

106195

## Slide 28
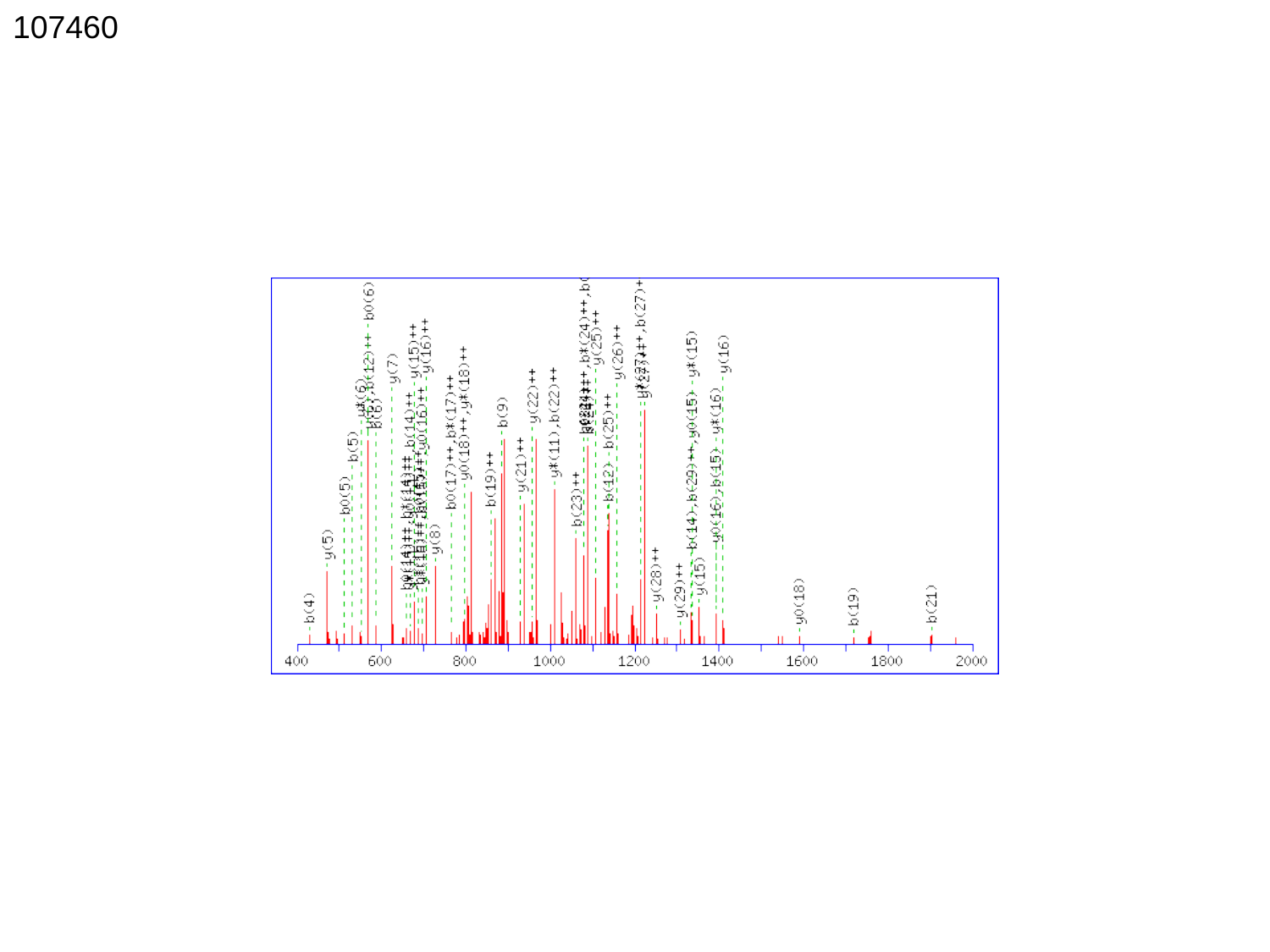

107460

## Slide 29
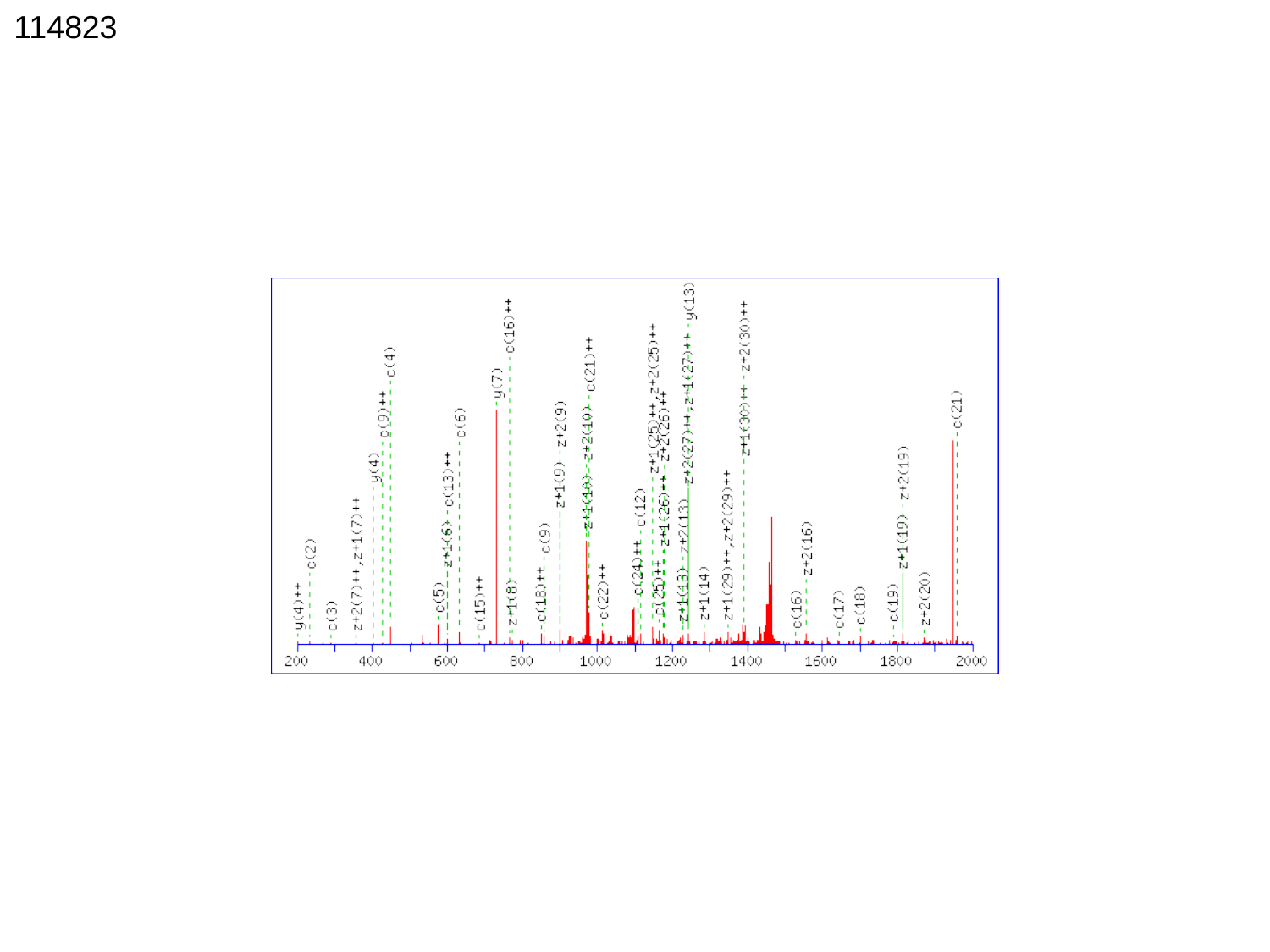

114823

## Slide 30
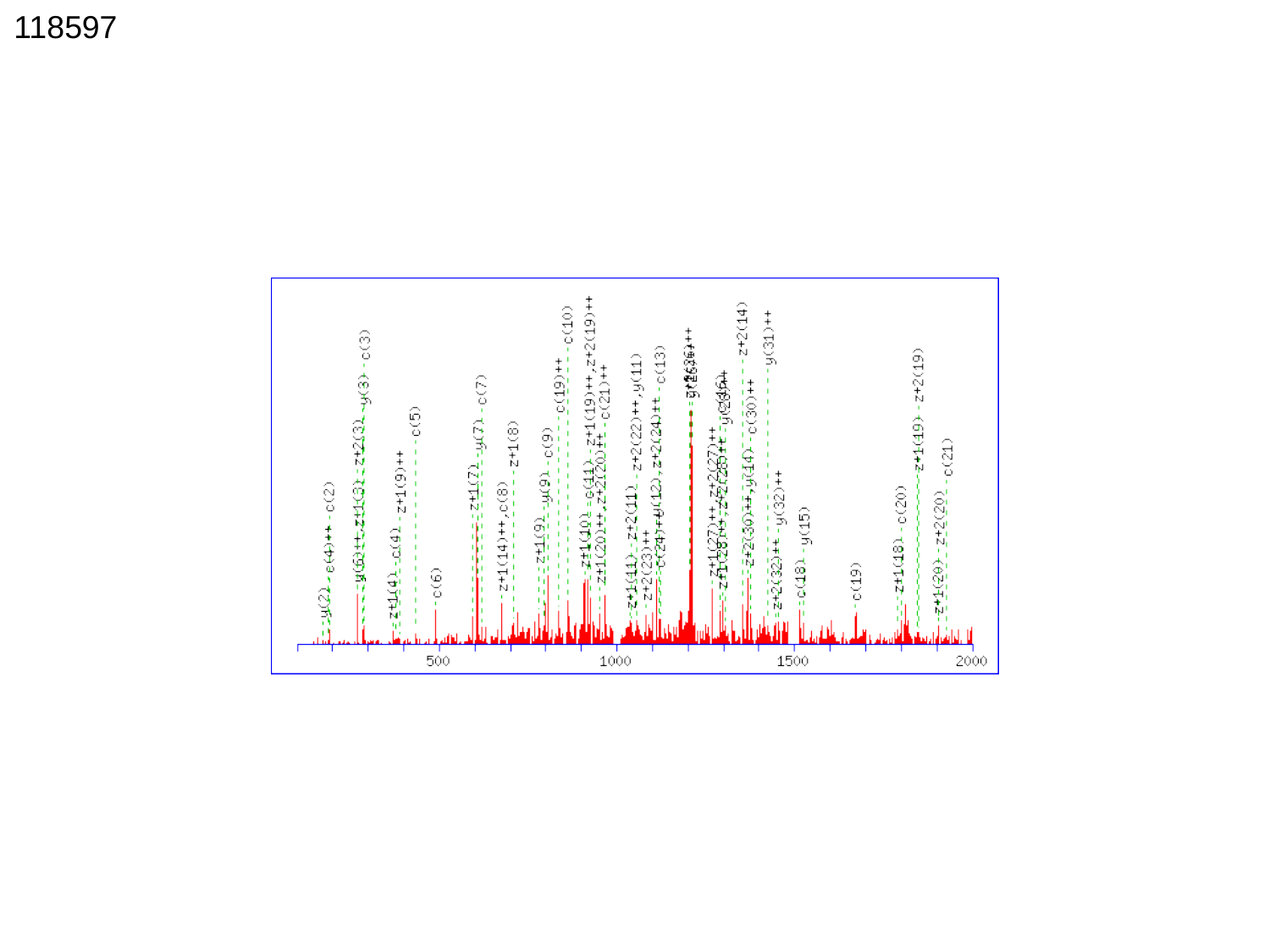

118597

## Slide 31
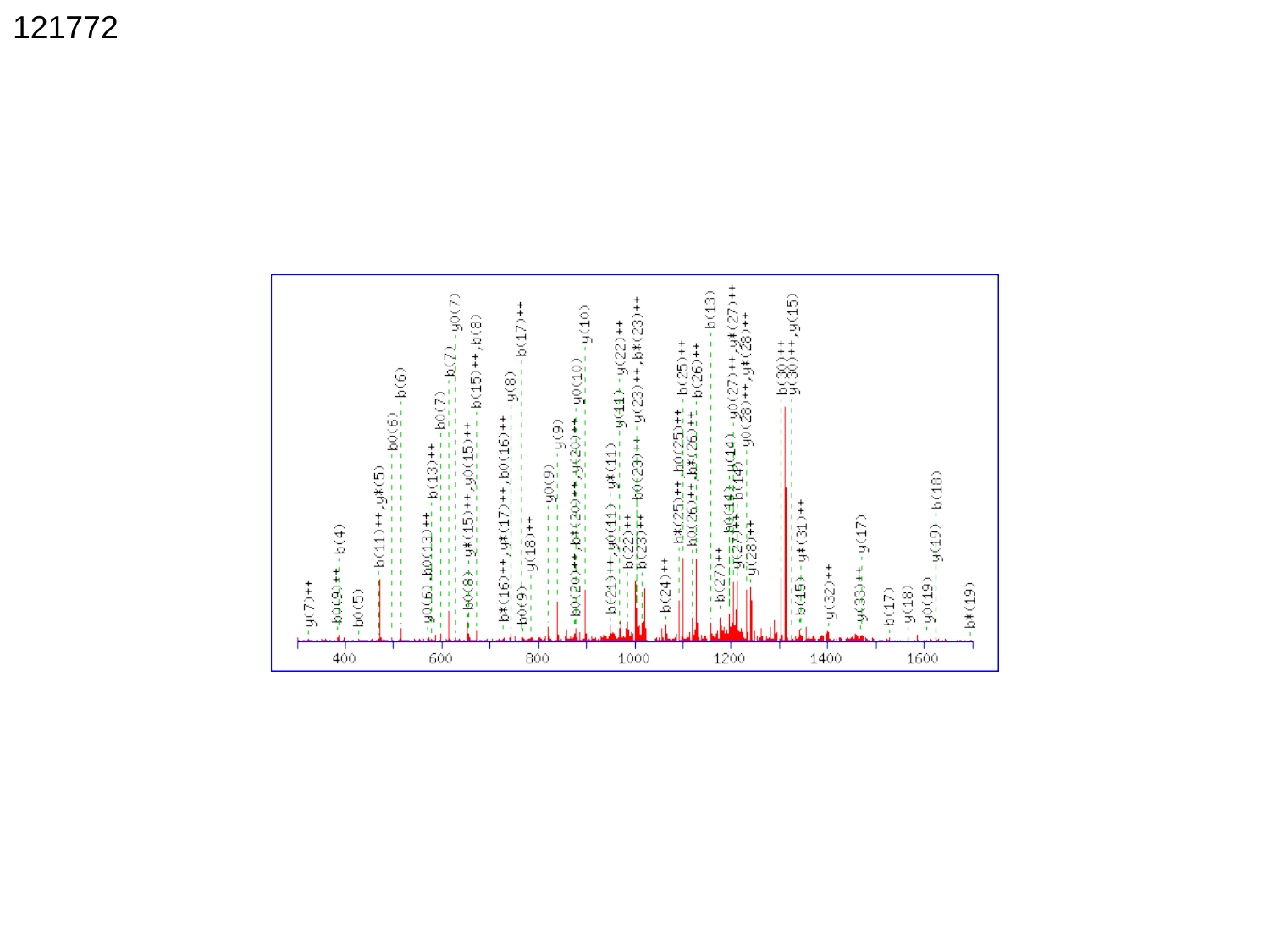

121772

## Slide 32
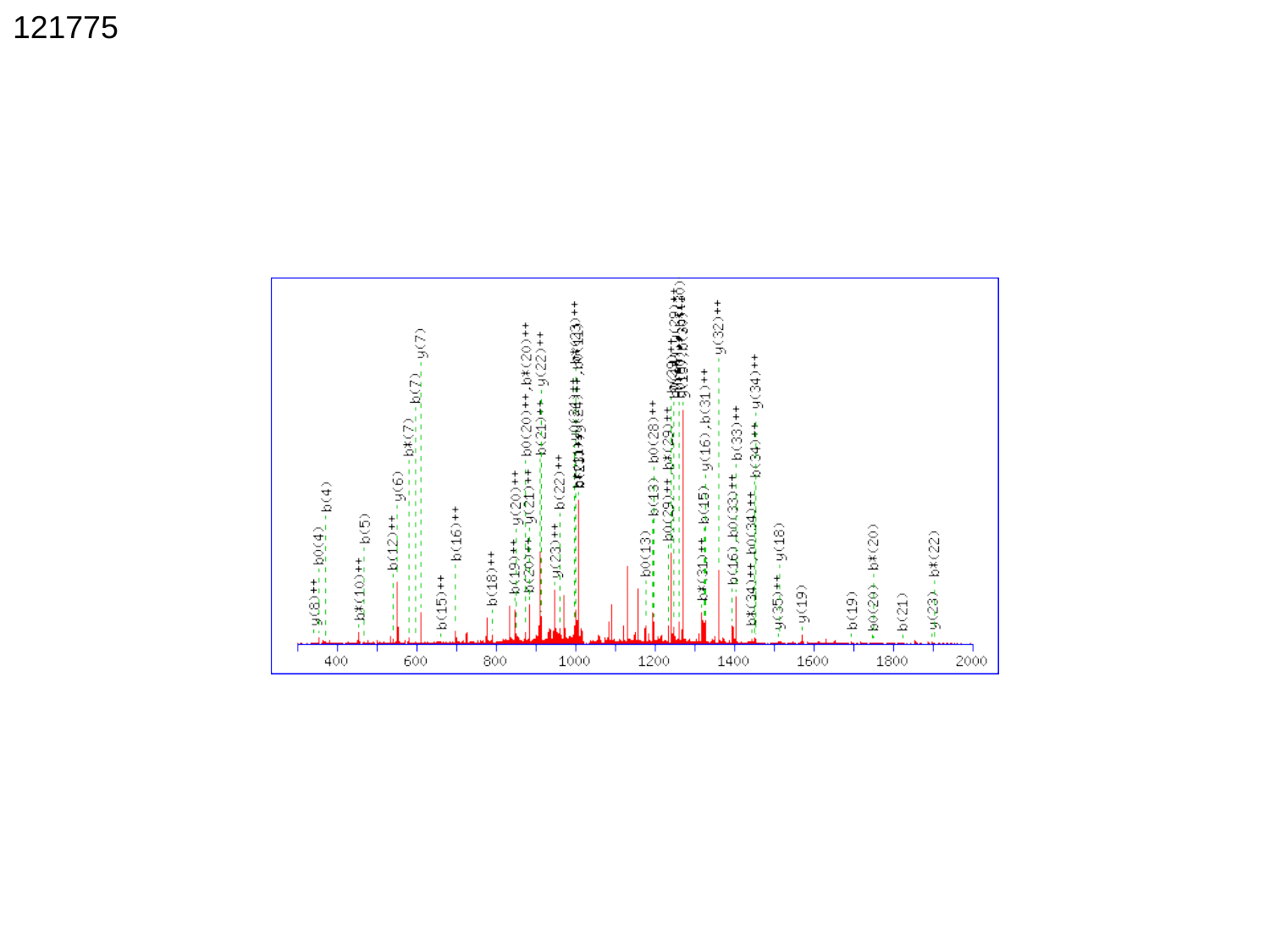

121775

## Slide 33
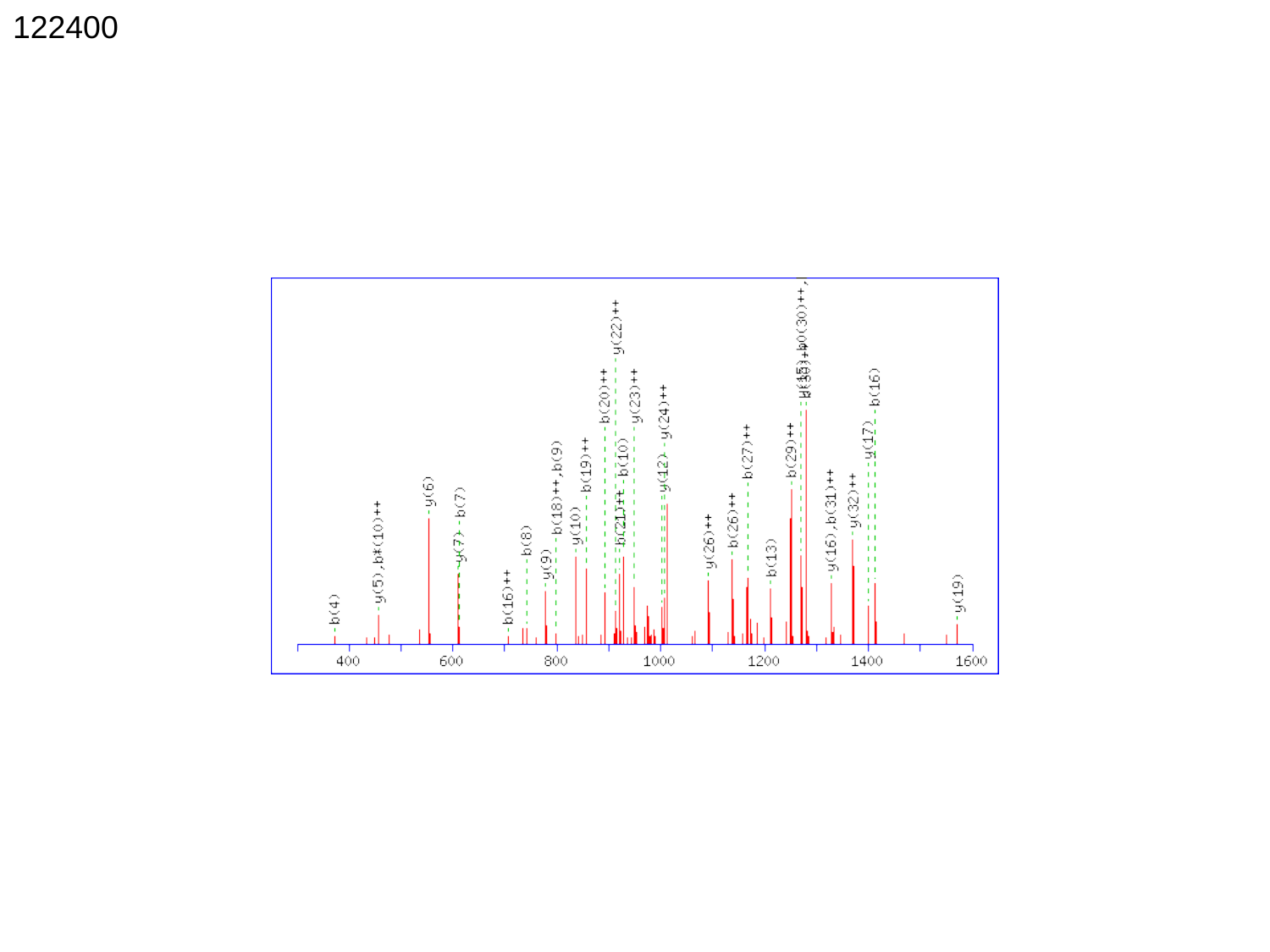

122400

## Slide 34
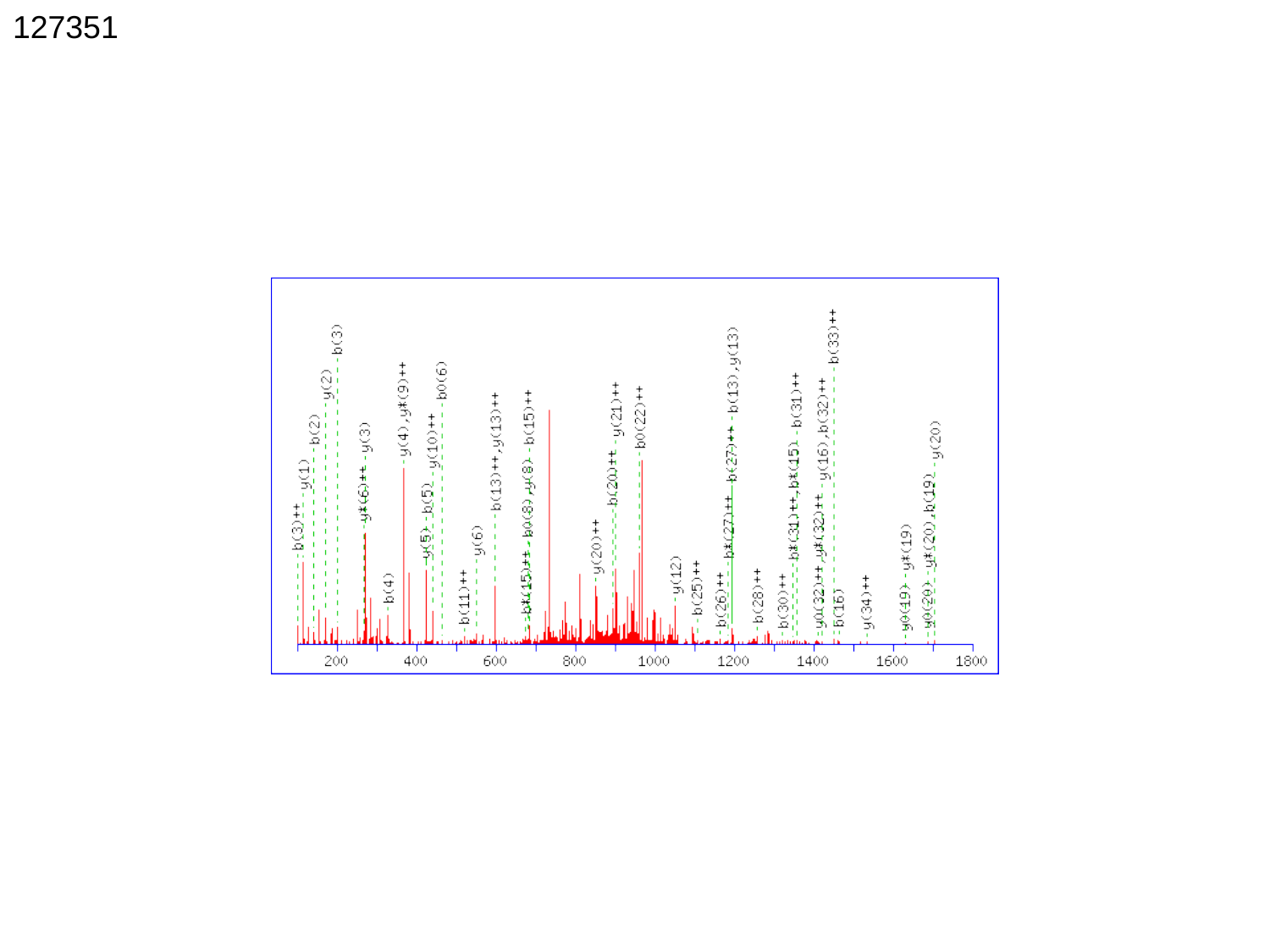

127351
